# Tau‑targeting multifunctional nanocomposite based on tannic acid-metal for near-infrared fluorescence/magnetic resonance bimodal imaging-guided combinational therapy in Alzheimer's disease

**DOI:** 10.7150/thno.98462

**Published:** 2024-09-30

**Authors:** Yutian Gu, Qin Zhang, Honglin Huang, Kwun Hei Willis Ho, Yu Zhang, Changqing Yi, Yifan Zheng, Raymond Chuen Chung Chang, Emma Shujun Wang, Mo Yang

**Affiliations:** 1 Department of Biomedical Engineering, The Hong Kong Polytechnic University, Hong Kong 999077, China.; 2 The Hong Kong Polytechnic University Shenzhen Research Institute, Shenzhen 518000, China.; 3 Research Center for Nanoscience and Nanotechnology, The Hong Kong Polytechnic University, Kowloon, Hong Kong 999077, China.; 4 Joint Research Center of Biosensing and Precision Theranostics, The Hong Kong Polytechnic University, Kowloon, Hong Kong 999077, China.; 5 Department of Electrical and Electronic Engineering, The University of Hong Kong, Hong Kong 999077, China.; 6 Department of Mechanical and Automotive Engineering, Royal Melbourne Institute of Technology, Melbourne VIC 3000, Australia.; 7 Guangdong Provincial Engineering and Technology Center of Advanced and Portable Medical Devices, School of Biomedical Engineering, Sun Yat-Sen University, Shenzhen 518107, China.; 8 Department of Neurology, The First Affiliated Hospital, Sun Yat-sen University, Guangdong Provincial Key Laboratory of Diagnosis and Treatment of Major Neurological Diseases, National Key Clinical Department and Key Discipline of Neurology, Guangzhou 510080, China.; 9 School of Biomedical Sciences, LKS Faculty of Medicine, The University of Hong Kong, Hong Kong 999077, China.

**Keywords:** Alzheimer's disease, tau pathology, oxidative stress, tannic acid, multifunctional nanocomposite

## Abstract

**Rationale:** Alzheimer's disease (AD) is hallmarked by amyloid-β (Aβ) plaques and hyperphosphorylated tau (p-tau) neurofibrillary tangles. While Aβ-centric therapies have shown promise, the complex pathology of AD requires a multifaceted therapeutic approach. The weak association between Aβ levels and cognitive decline highlights the need for alternative theranostic strategies. Currently, oxidative stress and tau hyperphosphorylation are now recognized as critical pathological events in AD. Thus, therapies that concurrently attenuate oxidative stress damage and inhibit tau pathology hold great potential for AD treatment.

**Methods:** Herein, a multifunctional neuron-targeted nanocomposite is devised to realize dual imaging-guided AD therapy, integrating the inhibition of tau pathology and reactive oxygen species (ROS)-neutralizing biofunctions. The construction of the nanocomposite incorporates polyphenolic antioxidants tannic acid (TA)-based nanoparticles carrying manganese ions (Mn^2+^) and fluorescent dye IR780 iodide (IR780), coupled with a neuron-specific TPL peptide. The resulting IR780-Mn@TA-TPL nanoparticles (NPs) are comprehensively evaluated in both *in vitro* and *in vivo* AD models to assess their imaging capabilities and therapeutic efficacy.

**Results:** The nanocomposite facilitates Mn-enhanced magnetic resonance (MR) imaging and near-infrared (NIR) fluorescence imaging. It effectively neutralizes toxic ROS and reduces tau hyperphosphorylation and aggregation. In AD rat models, the nanocomposite restores neuronal density in the hippocampus and significantly improves spatial memory.

**Conclusions:** Such a neuron‑targeting multifunctional nanocomposite represents a potential theranostic strategy for AD, signifying a shift towards bimodal imaging-guided treatment approaches.

## Introduction

Alzheimer's disease (AD) is a predominant neurodegenerative disorder and a leading cause of dementia worldwide. It is characterized by memory loss and cognitive decline. The etiology of AD includes the aggregation of amyloid-β (Aβ) plaques and the formation of neurofibrillary tangles (NFTs) caused by hyperphosphorylated tau protein 1, 2. Although significant efforts have been directed towards Aβ-centric interventions over the past few decades, the weak association between Aβ levels and cognitive decline highlights the urgent need to explore alternative therapeutic approaches 3-6. Accumulating evidence links tau hyperphosphorylation and aggregation with the exacerbation of cognitive deficits in AD. In the pathogenic milieu of AD, tau protein undergoes aberrant phosphorylation and then initiates a cascade of self-replicating protein aggregation that forms neurotoxic NFTs, resulting in neuronal injury and synaptic dysfunction 7. Furthermore, the dysfunction of tau protein leads to mitochondrial impairment and induces neuronal oxidative stress. Concurrently, during the asymptomatic prodromal phase of AD, there is a marked increase in reactive oxygen species (ROS) within the hippocampal and cortical regions, implicating oxidative stress may serve as a precursory event in AD pathogenesis 8-10. Excessive ROS prompts the accumulation of mitochondrial superoxide, leading to neuronal apoptosis. It also interferes with protein kinase B (AKT)/glycogen synthase kinase-3 beta (GSK3β) signaling pathway, thereby exacerbating tau hyperphosphorylation at multiple residues and resulting in neuronal damage, which consequently accelerates the progression of AD 11. Therefore, therapeutic approaches that simultaneously achieve ROS clearance and suppress tau pathology may provide feasible and effective treatments for AD.

Natural polyphenolic antioxidants are increasingly recognized for their potential in AD therapy, attributable to their widespread availability, good biocompatibility and potent antioxidative properties 12. Tannic acid (TA), a polyphenol comprised of a glucose core esterified with ten gallic acid units, stands out for its multifaceted therapeutic possibilities in AD 13-16. The dense hydroxyl groups of TA confer a robust capacity to neutralize deleterious ROS such as hydroxyl radical (•OH), hydrogen peroxide (H_2_O_2_), and superoxide anions (O_2_^•-^) 17, 18. Moreover, the polyphenolic structure of TA enables it to recognize the R3 peptide region of tau protein by forming hydrogen bond-mediated hairpin-like configurations, which is postulated to impede pathological conformational transitions and consequently mitigate the aggregation of neurotoxic tau filaments 19, 20. Additionally, the catechol moieties within TA facilitate strong chelating interaction with metal ions for magnetic resonance imaging (MRI) that could achieve non-invasive imaging of brain tissues in AD with a high spatial resolution 18, 21. Among MRI contrast agents, manganese (Mn) exhibits strong paramagnetism and significantly shortens the T1 relaxation time, making it an excellent candidate for MRI applications 22. Several works have employed Mn-enhanced MRI in preclinical animal models of neurodegenerative diseases through locally injected MnCl_2_ to enhance of the contrast in the brain 23, 24. However, Mn ions are relatively unstable in the physiological environment and rapidly distribute to various tissues, increasing the risk of acute toxicity 24. The chelation of TA with Mn ions forms a nanoagent that facilitates the delivery of Mn-based contrast agents while avoiding acute toxicity risks associated with free Mn ions. Therefore, TA is envisaged as a versatile candidate for developing Mn-enhanced MRI guided combinational therapies that concurrently address oxidative stress and tau pathology, representing a comprehensive approach to AD management. Yet, TA suffers from the inherent limitations such as poor physiological stability and rapid degradation, which prevent it from reaching efficacious concentrations within the cerebral milieu and thereby limit its therapeutic potential 25. Consequently, the development of an optimized drug delivery system is imperative to enhance the biostability and bioavailability of TA, thereby augmenting its therapeutic efficacy in the amelioration of AD.

The emergence of nanotechnology in drug delivery systems has significantly enhanced the therapeutic effects of natural compounds. In particular, the amphiphilic copolymer polyethylene glycol distearoylphosphatidylethanolamine (DSPE-PEG) is frequently employed in nanomedicine engineering due to its good biocompatibility and biodegradability, and it has been approved by the U.S. food and drug administration (FDA) for use in the medical field 26. DSPE-PEG-based nanocarriers are distinguished by their low critical micelle concentration (CMC), stable drug-loading capacity, and functional groups that are amenable to target-specific modification 27. Typically, the hydrophilic PEG forms an exterior shell that grants excellent dispersion of nanodrugs in water, while the hydrophobic DSPE creates an inner core suitable for encapsulating hydrophobic drugs. However, the hydrophilic nature of TA poses a challenge for encapsulation within DSPE-PEG nanocarriers using standard nanoprecipitation techniques 28. In our study, we address this challenge by aligning TA at the nanoparticle interface through hydrogen bonding with PEG 29-31, thus devising an alternative encapsulation strategy for the preparation of TA-based nanotherapeutics.

**Scheme 1:**
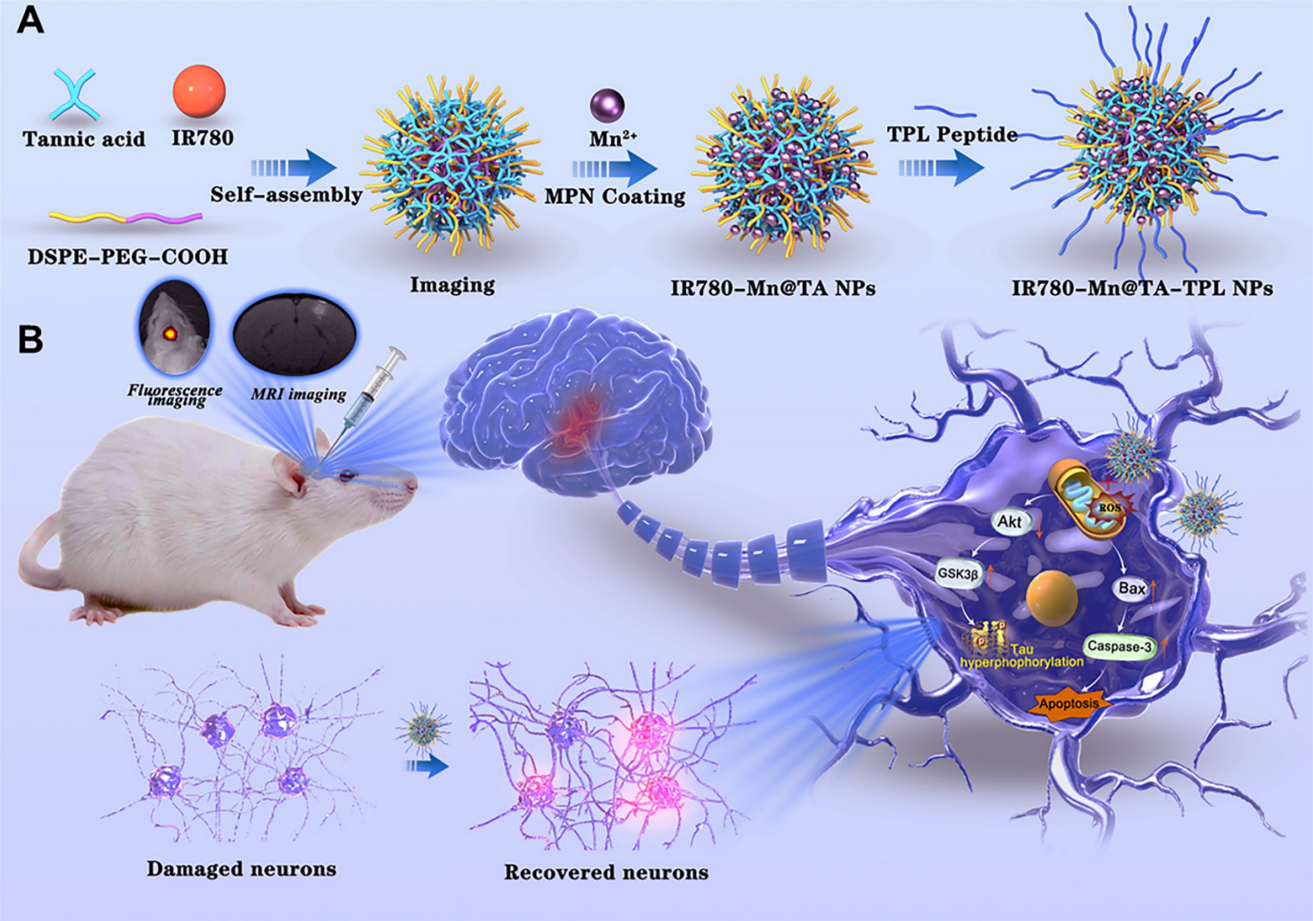
Schematic illustration of the preparation procedure of (A) IR780-Mn@TA-TPL NPs and (B) near-infrared fluorescence/magnetic resonance bimodal imaging-guided combinational therapy by IR780-Mn@TA-TPL NPs in AD model.

Herein, we report a TA-based multifunctional nanocomposite designed to achieve imaging-guided combinational therapy for AD (Scheme 1). The architecture of the nanocomposite utilizes the self-assembly of DSPE-PEG polymer molecules into nanomicelles with hydrophobic cavities that encapsulate IR780, a near-infrared (NIR) fluorescent dye that can be used for *in vivo* imaging due to its high photostability and minimal phototoxicity 32. Meanwhile, TA is strategically anchored on the nanocomposite surface to act as chelation sites for manganese ions (Mn^2+^), forming the Mn-containing IR780-Mn@TA NPs. Our strategic use of TA to chelate Mn^2+^ could avoid the risk of acute toxicity from excess free Mn ions and enables Mn-enhanced MRI in AD. To further tailor the nanocomposite for targeted AD therapy, we functionalized the NPs with a fusion peptide TPL (*l*-TGNYKALHPHNGGGGGHLNILSTLWKYR) 33 that comprises a blood brain barrier (BBB)-penetrating peptide TGN and a neuron binding peptide Tet1 through a four-glycine linker, yielding IR780-Mn@TA-TPL NPs (Scheme 1). This modification aims to enhance brain-neuron targeting, increase intracellular drug delivery, and mitigate off-target effects of the TA-based nanodrugs 34. The resulting IR780-Mn@TA-TPL NPs serve as a bimodal imaging agent, allowing for simultaneous NIR fluorescence imaging and MRI. The former aids in swiftly and effectively monitoring NPs accumulation at the lesion site via *in vivo* NIR FL imaging, thereby facilitating the investigation of dosage regimens to optimize the therapeutic efficacy of NPs 35, 36. The latter aspect endows the nanocomposite with the unique advantage of providing high-resolution anatomical information through MRI, allowing for detailed visualization of tissue structures and abnormalities within the brain. Moreover, IR780-Mn@TA-TPL NPs act as a therapeutic agent, facilitating multifaceted treatment modalities for AD. Our findings reveal that IR780-Mn@TA-TPL NPs exhibited excellent capability to (1) repair mitochondrial damage by reducing the accumulation of mitochondrial oxidative stress, (2) inhibit tau pathology through activating Akt/GSK-3β signaling pathway and attenuate tau hyperphosphorylation and toxic p-tau aggregation, and (3) prevent neuronal apoptosis via regulating pro-apoptotic proteins. These therapeutic actions collectively contribute to the recovery of neuronal density in the cortex and hippocampus and the amelioration of cognitive deficits in AD model rats. Consequently, these findings underscore the potential of our TA-Mn based neuron-targeting multifunctional nanocomposite for NIR fluorescence/MRI bimodal imaging-guided combination therapy as a viable strategy for AD treatment.

## Results and Discussion

### Preparation and characterization of IR780-Mn@TA-TPL NPs

**Figure 1:**
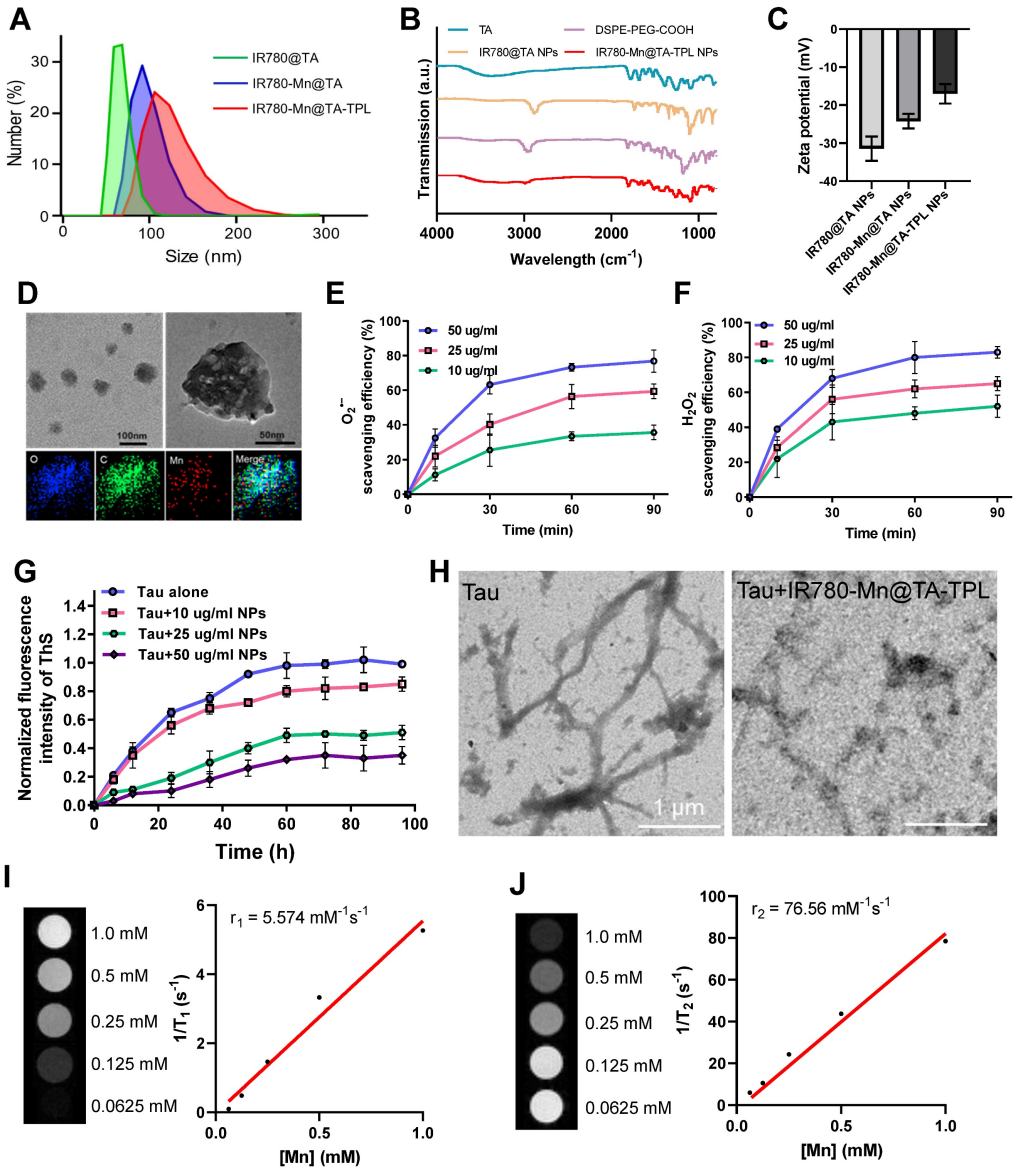
(A) Size distribution of NPs by DLS. (B) FT-IR spectra of TA, DSPE-PEG-COOH, IR780@TA NPs and IR780-Mn@TA-TPL NPs. (C) Zeta potential of NPs. (D) TEM images and EDS mapping of IR780-Mn@TA-TPL NPs. (E) O_2_^•-^ scavenging activity (%) and (F) H_2_O_2_ scavenging activity (%) of IR780-Mn@TA-TPL NPs (10, 25 and 50 μg/mL) at different time points. (G) Aggregation kinetics of tau protein monitored by ThS fluorescence upon addition of IR780-Mn@TA-TPL NPs at 37 °C. (H) TEM images of tau protein aggregation upon addition of IR780-Mn@TA-TPL NPs. (I) T_1_-weighted MR images of IR780-Mn@TA-TPL NPs (left). Plot of 1/T_1_ over Mn ion concentration of IR780-Mn@TA-TPL NPs; the slope indicates the specific reflexivity (r_1_) (right). (J) T_2_-weighted MR images of IR780-Mn@TA-TPL NPs (left). Plot of 1/T_2_ over Mn ion concentration of IR780-Mn@TA-TPL NPs; the slope indicates the specific reflexivity (r_2_) (right).

The fabrication process of IR780-Mn@TA-TPL nanocomposite is illustrated in Scheme 1A. Initially, DSPE-PEG-COOH polymer molecules underwent self-assembly to form micelles, encapsulating the fluorescent dye IR780 within their hydrophobic cores. Concurrently, TA was conjugated to the nanoparticle surface, yielding IR780@TA NPs. The transmission electron microscopy (TEM) image revealed the spherical assemblies of IR780@TA NPs with a hydrodynamic diameter of ~72 nm measured by dynamic light scattering (DLS) (Figure 1A and Figure S1A). The UV-vis spectrum of IR780@TA NPs showed a strong absorption peak near 780 nm, akin to that of free IR780 molecules (Figure S1B), suggesting successful encapsulation of IR780 in the nanosystem. The encapsulation efficiency (EE) was calculated to be 75.8% according to the standard absorbance curve of IR780 (Figure S2). Moreover, Fourier-Transform Infrared (FT-IR) spectrum revealed that the strength of the phenolic hydroxyl group of TA at 3334 cm^-1^ was significantly weakened upon the formation of NPs (Figure 1B and S3), which hinted at intermolecular hydrogen bonding between TA and the PEG segments 29, 30. In addition, the characteristic peak of PEG block, i.e., C-H stretching vibration at 2885 cm^-1^ was successfully observed in the spectrum of IR780@TA NPs (Figure 1B and S3). Subsequently, manganese ions (Mn^2+^) serving as a contrast agent for MR imaging were integrated into the nanocomplex via chelation with TA, resulting in the formation of IR780-Mn@TA NPs. X-ray photoelectron spectroscopy (XPS) analysis of O_1s_ spectra suggested the formation of O-Mn with a binding energy located at 530.26 eV, indicating the presence of coordination between Mn and the hydroxyl groups of TA in IR780-Mn@TA NPs (Figure S4) 37. Energy-dispersive spectroscopy (EDS) analysis also confirmed the successful introduction of Mn^2+^ within the nanocomposite (Figure S5). Meanwhile, chelation with Mn^2+^ led to a significant change in the surface charge of NPs, from -31.4 mV for IR780@TA to -23.9 mV for IR780-Mn@TA (Figure 1C). The neuron-targeting and BBB penetration capabilities of the nanocomplex were then endowed by surface functionalization with TPL peptide to form IR780-Mn@TA-TPL NPs. This was achieved through covalent linkage between the carboxylate groups present at the termini of PEG and the amine groups on the TPL peptide. The FT-IR spectrum of IR780-Mn@TA-TPL exhibited a new peak at 1672 cm^-1^, corresponding to the stretching vibration of amide bonds (Figure 1B and S3). To visualize TPL within the NPs, we labeled the peptide with fluorescein isothiocyanate (FITC), producing FITC-TPL. After the ultrafiltration centrifugation process to remove unincorporated FITC-TPL, the nanocomposite IR780-Mn@TA-TPL demonstrated significant intracellular green fluorescence from FITC-TPL and red fluorescence from IR780 upon cellular uptake of NPs. The colocalization coefficient was approximately 0.95, indicating successful modification with the TPL peptide (Figure S6). Morphologically, IR780-Mn@TA-TPL NPs appeared to be spherical, resembling sesame balls, with an average diameter of 116 nm (Figure 1A and D). The zeta potential of IR780-Mn@TA-TPL NPs was determined to be -16.5 mV, indicative of their suitability for *in vivo* applications (Figure 1C) 38. In addition, photostability assessment of IR780-Mn@TA-TPL NPs showed that they retained 61.8% of their absorbance after 24 h of light exposure, outperforming free IR780 molecules that nearly completely degraded under similar conditions (Figure S7A and B). Furthermore, IR780-Mn@TA-TPL NPs exhibited minimal change in average diameter after 7 days in phosphate-buffered saline (PBS), underscoring their strong colloidal stability in physiological solutions (Figure S7C).

Afterwards, we examined the antioxidant capacity of IR780-Mn@TA-TPL NPs by examining their ability to scavenge ROS, including O_2_^•-^, H_2_O_2_, and •OH. The NPs demonstrated a high ROS scavenging activity in a concentration-dependent manner, achieving maximal scavenging capacity after 1 h incubation. Notably, treatment with 50 μg/mL of the NPs resulted in scavenging approximately 75% of O_2_•-, 85% of total H_2_O_2_, and 70% of •OH (Figure 1E-F and Figure S8A). To further confirm the antioxidative properties of IR780-Mn@TA-TPL NPs, a free radical scavenging experiment was performed using the classic α,α-diphenyl-β-picrylhydrazyl (DPPH) assay 39. The results indicated that almost 90% of the free radicals were eliminated by a low concentration of IR780-Mn@TA-TPL NPs at 50 μg/mL (Figure S8B). These findings attest to the strong antioxidative efficacy of the nanocomposite, highlighting its prospective role in modulating oxidative stress for AD therapy.

We then investigated the inhibitory effect of IR780-Mn@TA-TPL NPs on pathological tau protein aggregation. The screening was based on the thioflavin S (ThS) fluorescence assay where the emission of ThS was strongly increased upon binding to β-sheet structures present in tau aggregates 11, 40. We measured the ThS fluorescence signal to monitor the aggregation of tau protein induced by heparin and the inhibition effects by various concentrations of our NPs 41. The results revealed that the fluorescence intensity of ThS in the presence of tau protein alone increased over time, displaying a sigmoidal growth curve characteristic of tau protein aggregation (Figure 1G). Notably, co-incubation of tau protein with IR780-Mn@TA-TPL NPs at a concentration of 50 μg/mL led to a significant reduction in maximal ThS fluorescence intensity to 32.5% compared to the control with tau protein alone (Figure 1G), thus confirming the inhibition of tau aggregation by our NPs.

Moreover, to further evaluate the inhibitory effect of the nanocomposite on tau fibrillogenesis, we observed the morphological changes of tau protein in the presence and absence of NPs via TEM images. After induction by heparin, tau protein samples without NPs exhibited numerous typical fibrils that extended several micrometers long, which was characteristic of the protein aggregation (Figure 1H). In contrast, tau protein treated with IR780-Mn@TA-TPL NPs exhibited a pronounced reduction in both the number and length of tau fibrils (Figure 1H), supporting the excellent efficacy of IR780-Mn@TA-TPL NPs in mitigating tau protein aggregation. To explain this inhibitory effect, the isothermal titration calorimetry (ITC) analysis was performed to evaluate the interaction of IR780-Mn@TA-TPL NPs with tau protein. The results demonstrated a high binding affinity between the IR780-Mn@TA-TPL NPs and tau aggregates with a dissociation constant (Kd) calculated to be 16.10 μM (Figure S9). This supported the effectiveness of our NPs in targeting tau aggregates and provided a rationale for their ability to inhibit tau aggregation.

In addition, we assessed the MR imaging capability of IR780-Mn@TA-TPL NPs. These NPs displayed an r_1_ relaxivity of 5.574 mM^-1^S^-1^ and an r_2_ relaxivity of 75.58 mM^-1^S^-1^ (Figure 1I and J). In comparison, the MnCl_2_ showed an r_1_ relaxivity of 3.808 mM^-1^S^-1^ and an r_2_ relaxivity of 69.02 mM^-1^S^-1^ (Figure S10). The r_2_/r_1_ ratio of IR780-Mn@TA-TPL NPs was lower than that of MnCl_2_, potentially due to the assembled state of Mn^2+^ within the nanosystem. Furthermore, the release rate of Mn in IR780-Mn@TA-TPL NPs was extremely slow, with only 3.16% and 3.95% Mn released after 4 days of storage in PBS and cell culture medium (Figure S11). Together, these findings illustrated the successful synthesis of a multifunctional nanocomposite IR780-Mn@TA-TPL NPs, showcasing remarkable antioxidant properties, the ability to suppress toxic tau pathology, strong photostability for NIR fluorescence imaging, and potential prospects for MR imaging applications.

### Specific neuron targeting ability of IR780-Mn@TA-TPL NPs

**Figure 2:**
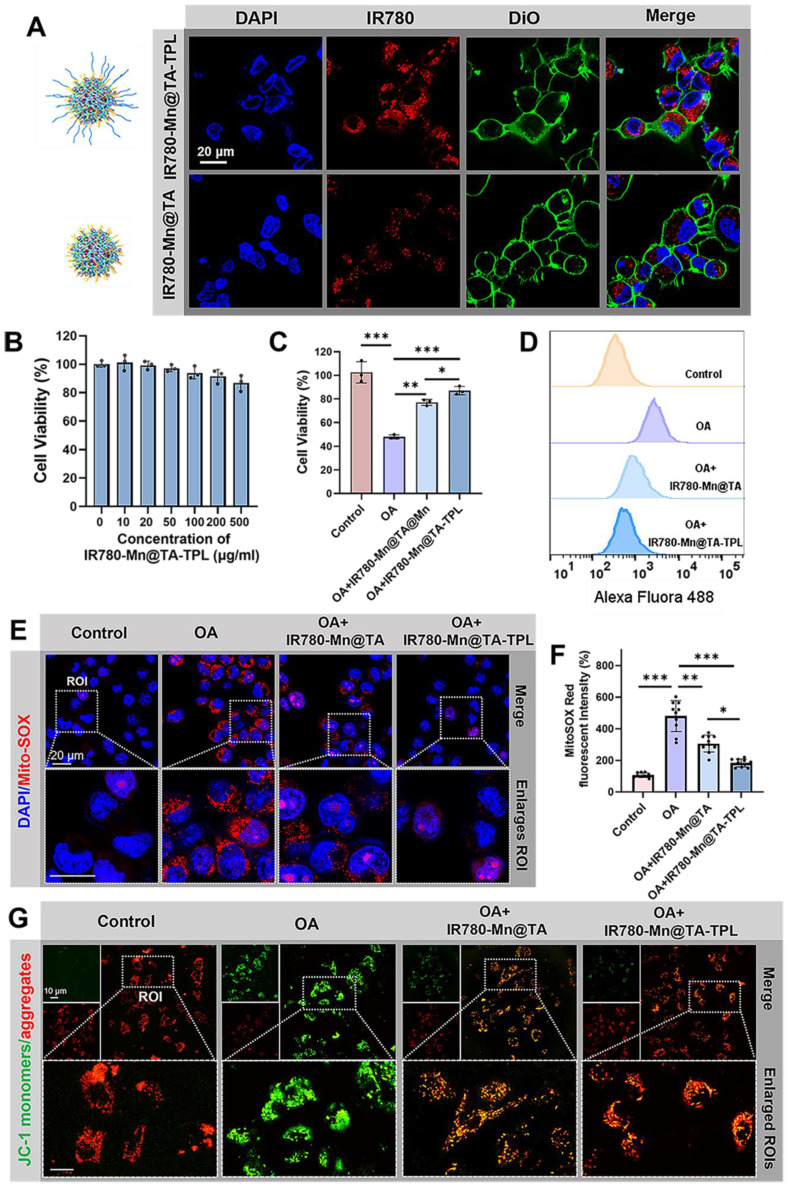
(A) Confocal microscopic images of PC12 cells after incubation with IR780-Mn@TA and IR780-Mn@TA-TPL NPs. DAPI: λex = 405, λem = 420-450 nm, red fluorescence of NPs: λex = 775 nm, λem = 790-820 nm. Scale bar: 20 μm. (B) The relative cell viability of PC12 cells after treated with IR780-Mn@TA-TPL NPs of different concentrations. (C) The relative cell viability of PC12 cells after incubation with OA, following by different treatments with NPs. (D) Quantitative analysis of intracellular ROS level indicated by the fluorescence intensity of DCFH-DA probe via flow cytometry assay. (E) Representative microscopic images of MitoSOX staining in PC12 cell after different treatments. Red MitoSOX: λex = 561 nm, λem = 575-625 nm. Scale bar: 20 μm. (F) Quantitative analysis of intracellular MitoSOX fluorescence intensity via ImageJ software. Data were presented as mean ± SD. Significance: ** p < 0.01 and *** p < 0.001. (G) Representative fluorescence images of OA-damaged PC12 cells after various treatment stained with JC-1 probe to evaluate the mitochondrial membrane potential. Green JC-1 monomers: λex = 488 nm, λem = 500-530 nm, red JC-1 aggregates: λex = 561 nm, λem = 575-625nm. Scale bar: 10 μm.

We verified the neuron-specific targeting ability of our nanoplatform by comparing the uptake of NPs between neurons (PC12 cells) and non-neuronal cells (BV2 cells). PC12 cells exhibited significantly brighter fluorescence after incubation with IR780-Mn@TA-TPL NPs than those exposed to IR780-Mn@TA NPs (Figure 2A). Quantitatively, the fluorescence intensity of PC12 cells incubated with IR780-Mn@TA-TPL NPs for 4 h was ~7.5-fold higher than that of cells treated with IR780-Mn@TA NPs (Figure 2A and Figure S12B). Conversely, BV2 cells, which are derived from murine microglia, showed no significant uptake difference between IR780-Mn@TA-TPL NPs and IR780-Mn@TA NPs (Figures S12A and C). Furthermore, Bio-TEM analysis provided visual evidence of the enhanced neuron-targeting potential of TPL peptide-containing NPs in PC 12 cells (Figure S13). These results indicated that TPL peptide modification selectively improved the affinity of the NPs for the neuron cells. In addition, we established an *in vitro* BBB transwell model (Figure S14A). Mouse brain microvascular endothelial (bEnd.3) cells were cultured on the upper layer for 7 days to form a monolayer layer of endothelial barrier (Figure S14B), and PC12 cells were seeded in the basolateral side 42. The integrity of the BBB model was monitored using FITC-conjugated dextran polymer (5, 10, and 100 kDa). The results showed that after 12 h incubation, only around 1.96% of 100 kDa dextran were able to cross the BBB, suggesting a tight cell-cell junction forming by the endothelium (Figure S14C). Subsequently, the NPs (IR780-Mn@TA-TPL or IR780-Mn@TA) were added into the apical side of the BBB transwell model. The results demonstrated that only a few IR780-Mn@TA NPs traversed to basolateral side and entered PC12 cells after 24 h, whereas the IR780-Mn@TA-TPL group exhibited significantly stronger fluorescence signals, ~3.5 times greater than those observed in IR780-Mn@TA treated cells (Figure S14D-F). The translocation rate of IR780-Mn@TA and IR780-Mn@TA-TPL group was quantified to be 4.1% and 13.8%, respectively (Figure S14G). These results indicated that TPL peptide modification facilitated NPs to penetrate through the upper endothelial layer and accumulated in neural cells.

### Mitigation of intracellular oxidative stress

We evaluated the cytotoxicity of as-prepared nanocomposite. The viability of PC12 cells remained above 87% following treatment with IR780-Mn@TA-TPL NPs for 12 h at a concentration of 500 µg/mL-a dosage 10-fold higher than that employed in the subsequent experiments (Figure 2B), suggesting minimal cytotoxicity toward neurons. Thereafter, we induced an AD cell model by subjecting PC12 cells to 50 nM okadaic acid (OA) for 12 h, which provoked excessive neuronal ROS production, along with abnormal tau protein hyperphosphorylation and aggregation 43. Cell viability assessments revealed that less than half of neurons survive after OA injury (Figure S15). However, the application of our nanocomposites significantly ameliorated this toxic effect, with the cell viability rising to 77.8% and 86.9% after incubation with IR780-Mn@TA NPs and IR780-Mn@TA-TPL NPs, respectively (Figure 2C).

We then investigated the intracellular ROS level using the fluorescent dye Dichlorodihydrofluorescein diacetate (DCFH-DA). OA exposure markedly augmented ROS levels by 5.8-fold compared to untreated control cells (Figure 2D and Figure S16A). Remarkably, a significant reduction in ROS overproduction was observed in IR780-Mn@TA NPs and IR780-Mn@TA-TPL NPs groups, with ROS level dropping to 2.6 and 1.7-fold of control cells, respectively (Figure 2D and Figure S16A). IR780-Mn@TA-TPL NPs outperformed IR780-Mn@TA NPs in scavenging intracellular ROS, which might be ascribed to the neuron-targeting capability conferred by TPL modification. This enhancement likely increased the cellular uptake of our nanocomplexes, thereby exerting therapeutic effects. Mitochondrial superoxide constitutes the primary ROS species contributing to neuronal oxidative stress and subsequent cell death 44. Thus, we employed MitoSOX probe to evaluate the capacity of our NPs to neutralize mitochondrial ROS accumulation. The findings indicated that over 80% of mitochondrial ROS were eliminated in OA-treated PC12 cells upon the introduction of IR780-Mn@TA-TPL NPs (Figure 2E and F), indicating the potent suppressive effect of these nanocomposites on mitochondrial oxidative stress injury. Additionally, mitochondrial membrane potential (ΔΨm) is one of the mitochondrial bioenergetic properties. Excess ROS generation can lead to mitochondrial dysfunction resulting in lower ΔΨm 45, 46. Hence, we evaluated ΔΨm using JC-1 dye that could form green-fluorescent monomers at low ΔΨm and red-fluorescent aggregates at high ΔΨm 47. OA-treated cells exhibited an increase in green JC-1 monomers and a concomitant decrease in red aggregates, with the red-to-green ratio of fluorescence intensity reduced to 38.8% relative to the untreated control (Figure 2G and Figure S16B). In contrast, IR780-Mn@TA NPs and IR780-Mn@TA-TPL NPs successfully restored the ΔΨm with red-to-green ratio of 63.5% and 79.5%, respectively (Figure S16B). The results confirm the effective repair of mitochondrial damage by our nanocomposite. Together, these findings support the strong antioxidant efficacy of IR780-Mn@TA-TPL NPs in neurons, providing a promising strategy to restore redox homeostasis in AD pathology.

### Inhibition of neuronal apoptosis and pathologic tau protein

**Figure 3:**
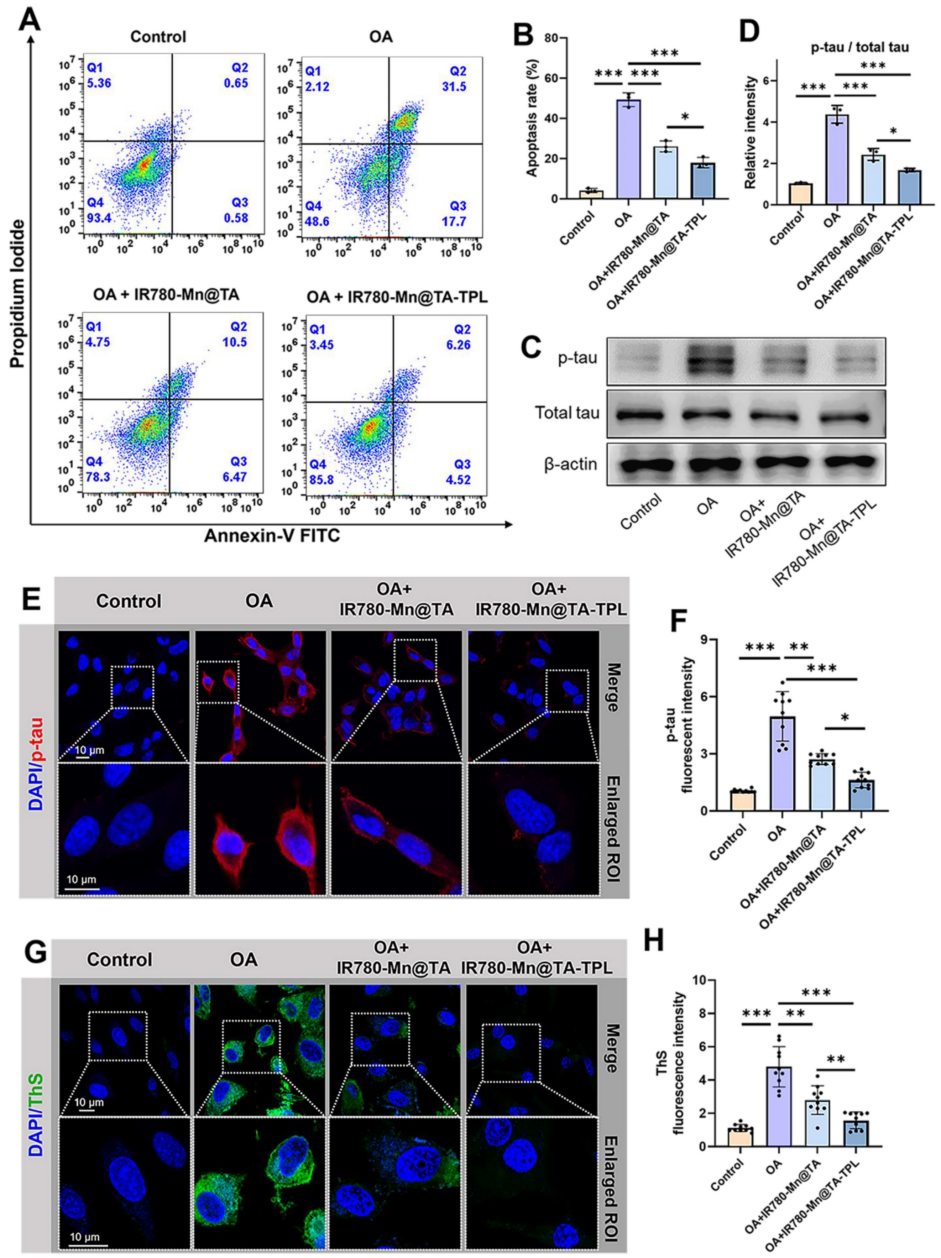
(A) The apoptosis rate of OA-damaged PC12 cells after incubation with NPs were measured by flow cytometry through PI/Annexin-V kit. (B) The quantification results of early and late apoptosis rate analyzed by Flowjo software. (C) Western blot images for p-tau and total tau protein expression in PC12 cells with different treatments. (D) Quantification of relative band intensity of p-tau / total tau by ImageJ software. (E) Representative fluorescence images of OA-damaged PC12 cells after various treatments, followed by staining with p-tau antibody. DAPI: λ_ex_ = 405, λ_em_ = 420-450 nm. Red p-tau: λ_ex_ = 561 nm, λ_em_ = 575-625 nm. Scale bar: 10 μm. (F) Quantification of fluorescent intensity of p-tau level using ImageJ software. (G) Representative fluorescence images of OA-damaged PC12 cells after various treatments stained with ThS to evaluate tau aggregates. Green ThS: λ_ex_ = 488 nm, λ_em_ = 500-530 nm. Scale bar: 10 μm. (H) Quantification of intracellular fluorescent intensity of ThS using ImageJ software. The data were presented as mean ± SD. Significance: *p < 0.05, ** p < 0.01, and *** p < 0.001.

As the restoration of redox homeostasis favors the reduction of neuronal apoptosis, we assessed cell apoptosis through flow cytometry using annexin V/propidium iodide-staining. As expected, the cell apoptosis rate of OA-damaged cells was 49.8% and this rate was markedly reduced to 13.5% in IR780-Mn@TA-TPL group, lower than 19.9% in IR780-Mn@TA group (Figure 3A and B). These findings underscore the superior efficacy of our nanocomposite in rescuing neurons from apoptosis. Our quantitative analysis of pathogenic p-tau protein indicated that treatment with our TA-based nanocomposite significantly downregulated OA-induced tau hyperphosphorylation (Figure 3C and D). Strikingly, IR780-Mn@TA-TPL NPs exhibited a more efficient inhibitory effect on tau hyperphosphorylation compared to NPs lacking TPL modification (Figure 3C and D). Further evidence was provided through immunostaining with the p-tau antibody, confirming that the treatment by IR780-Mn@TA-TPL NPs led to a significant reduction in neuronal p-tau expression (Figure 3E and F). In addition, we investigated the level of neuronal tau aggregates through ThS staining assay. We observed that the ThS fluorescent signal in OA-treated cells was much stronger than that in the untreated ones, but it became significantly weaker in the presence of IR780-Mn@TA NPs and IR780-Mn@TA-TPL NPs due to the inhibition of tau-tau binding (Figure 3G and H), demonstrating their high efficiency to prevent extensive tau assemblies. Collectively, our findings highlight the multifaceted therapeutic potential of IR780-Mn@TA-TPL NPs in AD by mitigating aberrant tau phosphorylation at critical sites and substantially reducing the formation of neurotoxic tau aggregates.

### Regulation of apoptosis-relevant proteins and activation of Akt/GSK3β signaling pathway

**Figure 4:**
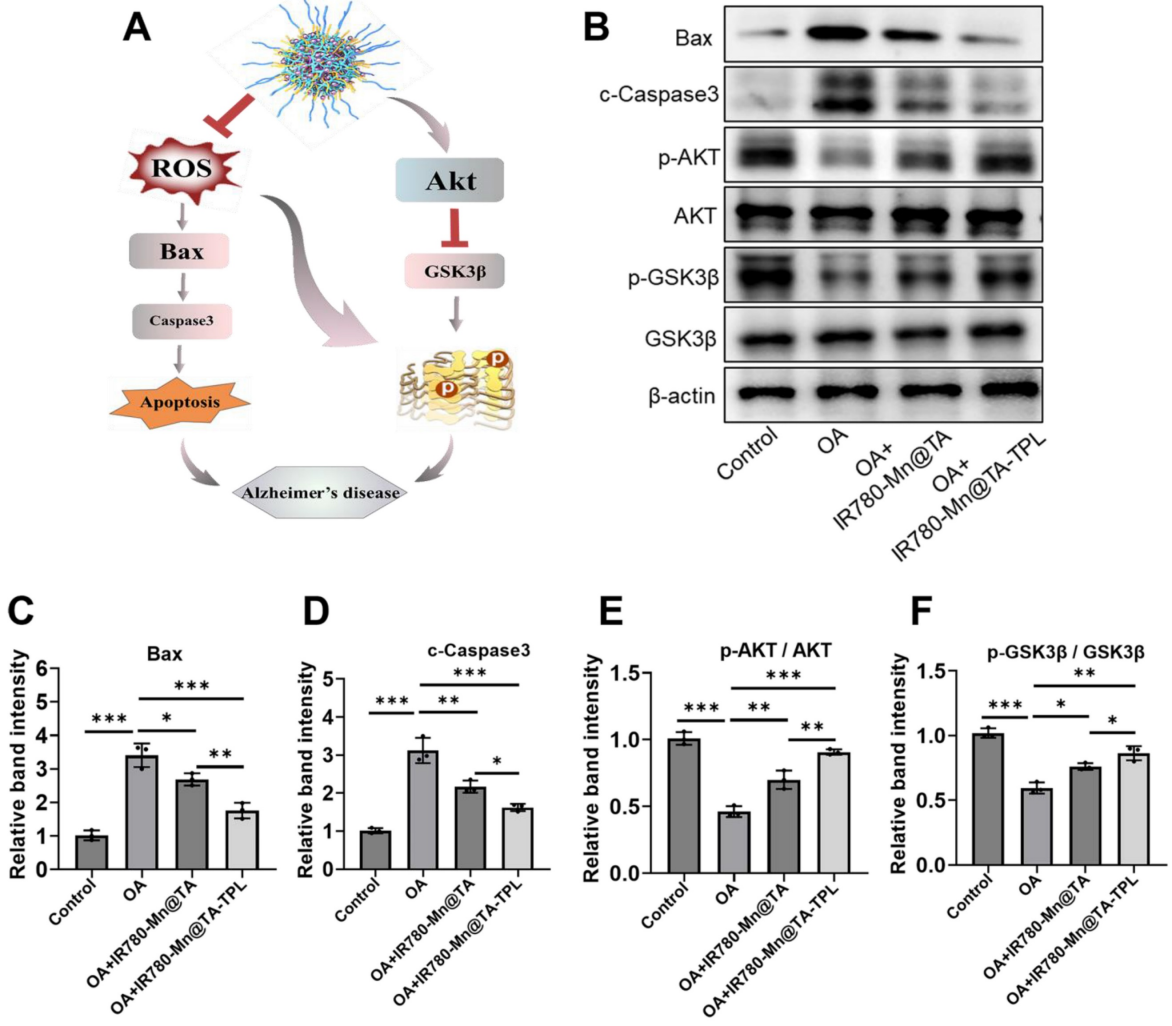
*In vitro* therapeutic mechanism of IR780-Mn@TA-TPL NPs. (A) Proposed model illustrating the mechanism of IR780-Mn@TA-TPL NPs in anti-apoptosis and the amelioration of tau hyperphosphorylation in OA-induced AD cell model. (B) Representative western blot images of Bax, c-Caspase3, p-Akt (p-Ser473), total AKT, p-GSK3β (p-Ser9), total GSK3βand in PC12 cells after different treatments. Quantification results of the band intensity of (C) Bax, (D) Caspase3, (E) p-Akt / AKT, and (F) p-GSK3β / GSK3β in the western blot assay. Data were presented as mean ± SD (n = 3). Significance: * p < 0.05, ** p < 0.01 and *** p < 0.001.

We further explored the underlying mechanism of IR780-Mn@TA-TPL NPs to prevent neurons from tau hyperphosphorylation and apoptosis. It is known that oxidative stress can induce apoptosis by activating the pro-apoptotic BCL2-associated X (Bax) and the downstream caspase-3 protein (Figure 4A) 48. Consequently, the relative level of Bax and caspase-3 can indicate whether the cells have launched the apoptotic cascade. Consistent with previous studies, the expression of Bax and caspase 3 was increased in OA-induced cells (Figure 4B-D) 11. Then, treatment with IR780-Mn@TA and IR780-Mn@TA-TPL NPs remarkably reduced Bax and cleaved caspase 3 (c-Caspase 3) expression, demonstrating that our nanocomposites can block the pro-apoptotic pathway (Figure 4B-D).

In addition, tau pathogenesis in AD involves two key serine/threonine-specific protein kinases, protein kinase B (AKT) and its downstream kinase glycogen synthase kinase 3β (GSK3β) 49. Our results showed that OA damage downregulated the expression of p-Akt (p-ser473) and p-GSK3β (p-ser9), indicating the inactivation of AKT/GSK3β signal pathway in OA-induced AD cell model (Figure 4B, E and F). Notably, the expression of p-Akt and p-GSK3β was found to be restored after treatment with IR780-Mn@TA NPs and IR780-Mn@TA-TPL NPs, indicating the nanocomplex attenuated tau pathology through activating Akt/GSK3β signaling pathway (Figure 4B, E and F). Therefore, our TA-based nanocomposite could protect neuronal cells against apoptosis by reducing pro-apoptotic proteins and inhibiting intracellular tau hyperphosphorylation through the activation of the Akt signaling pathway.

### *In vivo* imaging of IR780-Mn@TA-TPL NPs on the AD rat model

**Figure 5:**
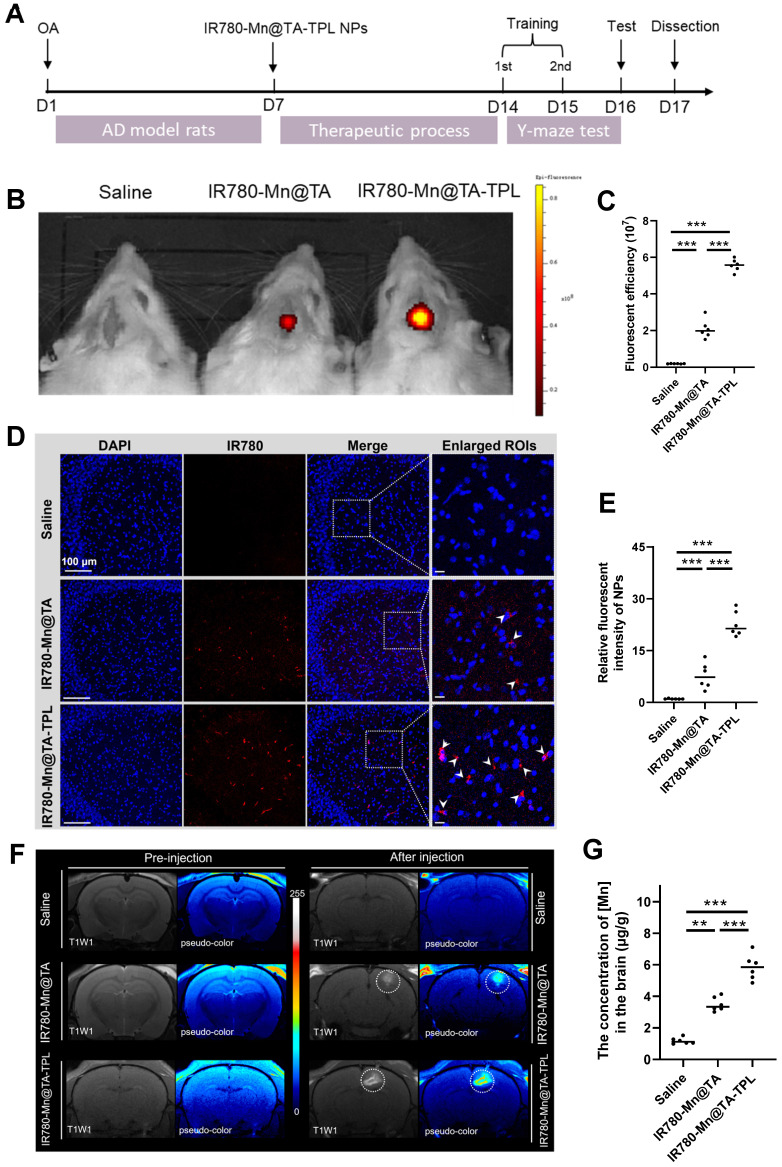
(A) Animal experiment flowchart. (B) Representative IVIS images of rats after administration of saline, IR780-Mn@TA and IR780-Mn@TA-TPL NPs. (C) Quantitative analysis of the average fluorescent intensity of NPs in the brain area by Living Imaging software. (D) Representative fluorescence images of the hippocampus region in the OA-treated rats after injection with NPs. Scale bar: 100 μm. (E) Quantitative analysis of the average fluorescent intensity of the NPs in the hippocampus regions of the brain sections. (F) *In vivo* T1-weighted MR images of the brain of OA-treated rats before and after administration of saline, IR780-Mn@TA NPs, and IR780-Mn@TA-TPL NPs. (G) Concentration of Mn^2+^ in hippocampus tissues was measured by ICP-MS. Significance: *** indicating p < 0.001, ** indicating p < 0.01 and * indicating p < 0.05 (n=6 per group). Error bars represent standard deviation.

We established an *in vivo* AD model by intracerebral administration of OA into rats to induce oxidative stress damage and tau hyperphosphorylation within the hippocampus (Figure 5A) 11, 50, 51. After 7 days of induction in AD model, we evaluated the neuron-targeting ability *in vivo* by monitoring accumulated fluorescence signals of IR780-Mn@TA-TPL in the brain (Figure 5B).

*In vivo* imaging (IVIS) results revealed that the fluorescence intensity in the brain regions post-injection of IR780-Mn@TA-TPL NPs was almost 3-fold higher than that observed in the IR780-Mn@TA group, indicating the superior neuron-targeting capability and longer neuronal retention of the TPL-decorated nanocomposite (Figure 5B and C). It was subsequently confirmed by the intense fluorescence detected in the hippocampal region of coronal brain sections from IR780-Mn@TA-TPL group (Fig. 5D and E). Notably, the fluorescence clusters of IR780-Mn@TA-TPL NPs (red) showed significant overlap with ThS-stained tau aggregates (green) in the brain sections of AD rats (Figure S17). This high degree of co-localization between NPs and Tau aggregates demonstrated the Tau aggregation-targeted molecular imaging capability of our probes.

Furthermore, 24 h after intravenous injection of NPs, the fluorescence intensity in the brain of rats treated with IR780-Mn@TA-TPL was significantly higher than that in the IR780-Mn@TA group (Figure S18A and B). The *ex vivo* fluorescence imaging revealed that IR780-Mn@TA-TPL NPs exhibited extensive distribution in the brain area compared to IR780-Mn@TA (Figure S18C and D). These results highlighted that TPL-modified approach increased the BBB-penetration efficacy of the nanocomplex. Moreover, we examined the ability of the nanocomplex for *in vivo* MR imaging. T1-weighted MR images were acquired before and after the injection of nanocomposites into the AD rats. The IR780-Mn@TA-TPL group exhibited a significantly stronger MR signal compared to IR780-Mn@TA group, suggesting the improved brain retention (Figure 5F). In addition, inductively coupled plasma mass spectrometry (ICP-MS) analysis of Mn^2+^ concentrations in brain tissue further demonstrated that IR780-Mn@TA-TPL NPs have superior retention (Figure 5G), potentially attributable to their specific neuronal binding performance.

### *In vivo* therapeutic outcomes of IR780-Mn@TA-TPL NPs on the AD rat model

**Figure 6:**
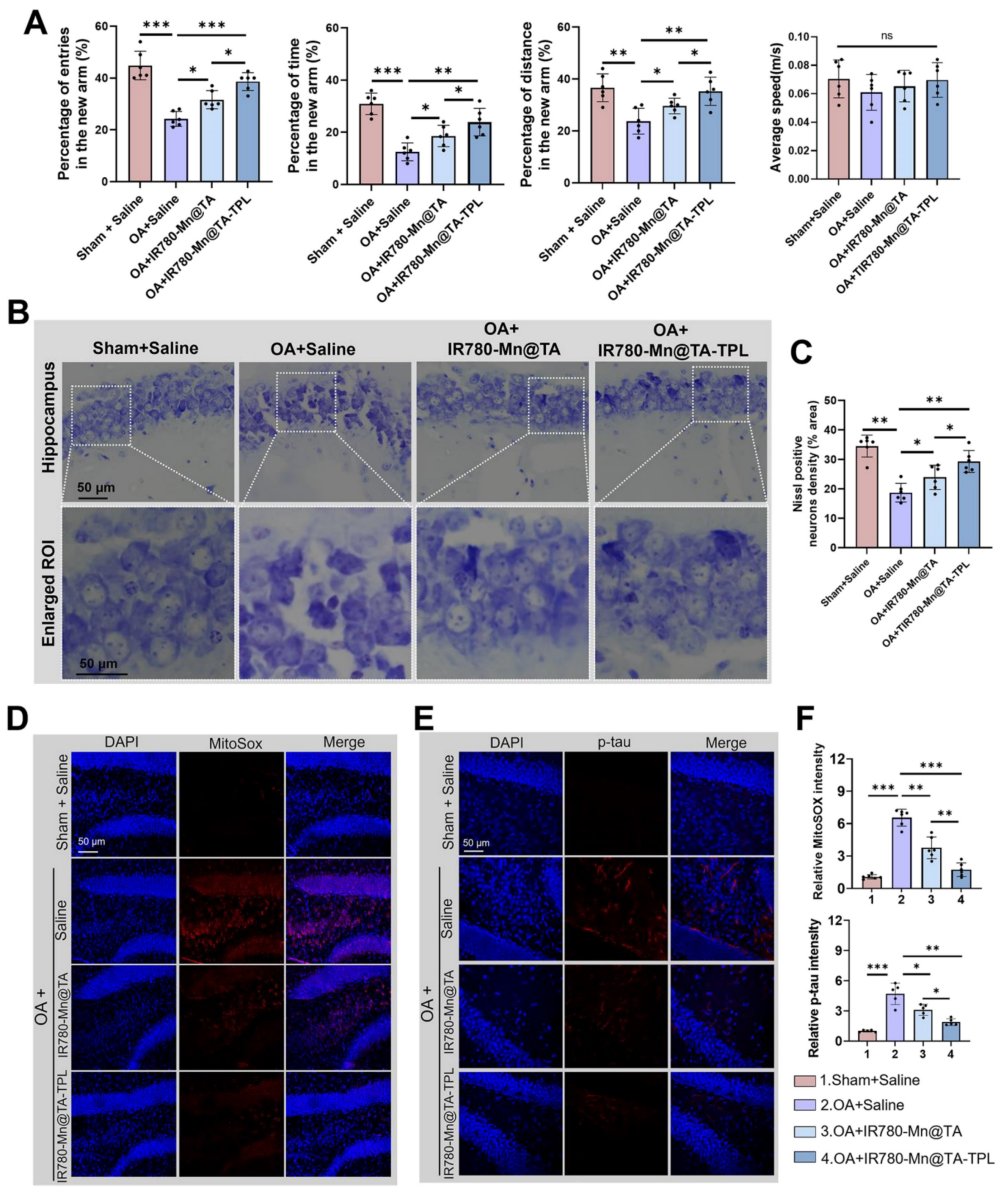
(A) Quantitative analysis on the percentage of entries, time, distance to the novel arm and average speed during the test phase in Y maze test. (B) Nissl staining analysis of the density and activity of neurons in the hippocampus. (C) Quantitative results of nissl-positive neuron density in the hippocampus. Representative confocal fluorescent images of brain slides stained with (D) MitoSOX and (E) p-tau. (E) Quantification of fluorescent intensity of MitoSOX and p-tau in (D) using ImageJ software. Data were presented as mean ± SD. Significance: *p < 0.05, ** p < 0.01 and *** p < 0.001.

Progressive cognitive deficits and memory loss are hallmark symptoms of AD. Therefore, we introduced the Y-maze behavior test to explore spatial learning and memory in AD rats by recording the frequency and duration they spent in the new arm 52, 53. OA-induced AD rats exhibited notably fewer entries and covered less distance within the new arm, only half as many as the sham controls (Figure 6A). Additionally, the time AD rats spent exploring the new arm was a mere 12.1% of the total, markedly lower than the 31.5% observed in their non-pathological counterparts, highlighting a severe deficit in spatial working memory after OA modeling (Figure 6A). Notably, subsequent treatment with IR780-Mn@TA-TPL NPs yielded a substantial increase in both entries and distance traveled by the AD rats, almost aligning them with sham control levels (Figure 6A). Furthermore, IR780-Mn@TA-TPL NPs-treated rats spent 25.8% of their time in the new arm, demonstrating a significant improvement compared to untreated AD rats (Figure 6A), indicative of ameliorated learning and memory functions. Nevertheless, mean locomotion speed remained almost consistent across groups (Figure 6A), implying no discernible effect of either OA or the nanocomposites on the motor functions of rats. These findings highlighted the great potential of the nanocomposite in mitigating AD-associated cognitive impairment.

We further performed nissl staining to examine the pathological morphology of neurons in the hippocampus, a region integral to spatial memory and tau pathogenesis in AD pathology. Compared to heathy sham counterparts, the nissl body-stained neurons were atrophied in OA-induced AD rats (Figure 6B). In contrast, following administration with our NPs resulted in more closely arranged neurons and increased neuronal integrity in hippocampal areas (Figure 6B). Quantitative analysis revealed that nissl-positive neuronal density increased to 24.2% and 29.8% in IR780-Mn@TA and IR780-Mn@TA-TPL groups respectively, higher than 17.5% measured in OA-damaged AD rats (Figure 6C). These findings support the *in vivo* neuroprotective efficacy of our nanocomposite.

Additionally, we evaluated the mitochondrial ROS levels in the cerebral areas using MitoSOX probe. Strikingly, ROS levels in the AD rat model were amplified by almost 6.5 folds compared to the sham counterparts (Figure 6D(i) and E(i)). A notable ROS scavenging effect was observed in AD rats after a 7-day treatment regimen with our nanocomposite. Specifically, IR780-Mn@TA-TPL NPs treatment demonstrated a more pronounced reduction in ROS levels, decreasing to nearly 1.8-fold above sham controls, compared to a 3.7-fold reduction observed with IR780-Mn@TA NPs (Figure 6D(i) and E(i)). This *in vivo* ROS attenuation trend aligned with those in the *in vitro* study.

Moreover, we conducted an immunofluorescence assay to evaluate the expression of p-tau in brain tissue. The results demonstrated p-tau was virtually undetectable in sham rats, whereas OA-induced AD rats displayed intense punctate staining, indicating a fluorescent intensity approximately 4.5 times greater than that of the sham controls (Figure 6D(ii) and E(ii)). The microinjection of our NPs (IR780-Mn@TA and IR780-Mn@TA-TPL) significantly mitigated p-tau expression. Notably, the fluorescent signal of p-tau in the hippocampus of IR780-Mn@TA-TPL group was only ~1.9 times than those in sham group, suggesting over half of the p-tau protein was inhibited by our nanocomposite (Figure 6D(ii) and E(ii)). Furthermore, apparent ThS-positive neurons were detected in the hippocampus areas in AD rats, indicating the presence of tau aggregates. Nevertheless, these fluorescent clusters nearly disappeared upon treatment with IR780-Mn@TA-TPL NPs (Figure S19), implying that our TA-based nanocomposite could efficiently mitigate the formation of toxic tau aggregates in neurons. Taken together, these histological analyses strongly suggest that our NPs effectively neutralize ROS overproduction in neuronal cells, thereby preventing tau hyperphosphorylation in AD pathology.

### *In vivo* biosafety of IR780-Mn@TA-TPL NPs

To evaluate the* in vivo* biosafety of our engineered nanomaterials, we assessed ROS level in the brain tissues of wild-type rats after administration of IR780-Mn@TA-TPL NPs. The results of mitoSOX and DCFH-DA staining showed no significant positive signals in the brain sections of both the saline-injected control and the NPs-injected group (Figure S20), suggesting the IR780-Mn@TA-TPL NPs did not cause ROS overexpression in the brain. These findings indicated that the administration of these Mn-containing NPs did not induce toxic oxidative stress in the rat. Additionally, we performed hematoxylin and eosin (H&E) staining on major organs (heart, liver, spleen, lungs, and kidneys) from rats administered with IR780-Mn@TA-TPL NPs (Figure S21). Compared to the wild-type control, animal injected with NPs showed no clear histopathological lesions. Furthermore, the hemolysis assay revealed that no hemolysis phenomenon occurred in NPs co-incubation groups (Figure S22), indicating the excellent blood biocompatibility of IR780-Mn@TA-TPL NPs. Altogether, the developed multifunctional nanocomposite had good biosafety for *in vivo* applications.

## Conclusions

In summary, we have developed a TA-derived neuron-specific nanocomposite that achieved dual imaging modality-guided combinational therapy in AD. By capitalizing on the inherent antioxidant properties of TA, the nanocomposite serves as a robust ROS scavenger and tau pathology inhibitor. The addition of the surface-decorated TPL peptide significantly enhances neuronal targeting and BBB penetration, thus augmenting the neuroprotective efficacy of the nanosystem. Additionally, the incorporation of Mn^2+^ and NIR dye IR780 endows the nanocomposite with dual functionality for both MR and NIR fluorescence imaging. Our findings demonstrated that IR780-Mn@TA-TPL nanocomposite effectively mitigates oxidative stress, diminishes tau hyperphosphorylation, and inhibits subsequent tau aggregation in AD model. Notably, the nanocomposite promotes significant recovery of neuronal density in the hippocampus and markedly ameliorates spatial memory deficits in AD rats. We believe that this neuron-targeted multifunctional nanocomposite heralds new therapeutic avenues for AD management.

## Materials and Methods

### Fabrication of IR780-Mn@TA-TPL NPs

To synthesize the IR780@TA NPs, DSPE-PEG-COOH (1 mL, 50 µg/mL), TA (1 mL, 50 µg/mL), and IR780 (1 mL, 10 µg/mL) were dissolved in dimethyl sulfoxide (DMSO) and subjected to stirring overnight. The subsequent mixture was then dropped into 20 mL of phosphate-buffered saline (PBS) and dialyzed against deionized (DI) water for a duration of 24 h using a Micro Dialysis System (MWCO 50 kDa). The resulting IR780@TA NPs were lyophilized and redispersed in water for the following experiments and storage. The encapsulation efficiency (EE%) of IR780 in NPs was determined by the following formula: EE%=(Total amount of IR780 molecules -The amount of free IR780 molecules in the supernatant after loading)/ Total amount of IR780 molecules × 100%. Here, the amount of IR780 molecules was determined by the standard absorbance curve of free IR780 in DMSO in UV-vis absorbance spectroscopy.

For peptide conjugation, the TPL peptide (*l*-TGNYKALHPHNGGGGGHLNILSTLWKYR) 33 was attached by activating carboxyl groups on the nanoparticle surface. Specifically, 2-[N-morpholino]ethane sulfonic acid (MES) buffer (3.9 mg/mL, 1 mL), 1-ethyl-3-(3-dimethylamino)propyl carbodiimide (EDC) (10 mg/mL, 1 mL), and N-hydroxysuccinimide (NHS) (2.5 mg/mL, 1 mL) were introduced to the nanoparticle solution and the mixture was incubated at 4 °C for 2 h. Subsequently, TPL peptide (1 mg/mL, 1 mL) was added to the activated nanoparticle solution and the reaction was allowed to proceed overnight at room temperature. The final product, IR780-Mn@TA-TPL NPs, was purified using ultrafiltration and subsequently lyophilized.

### Antioxidant activity evaluation

The antioxidant capacity of NPs was assessed at three different concentrations (10, 25, and 50 μg/mL) using the DPPH assay 18. To each mixture containing 500 μL of methanol and 1050 μL of an aqueous dispersion of IR780-Mn@TA-TPL NPs, 200 μL of a 0.35 mM DPPH solution (Sigma-Aldrich) in methanol was added. The final DPPH concentration in each sample was 50 μM. Absorbance measurements at 517 nm were measured using a UV-vis spectrophotometer (Ultrospec 2100 pro) both before and after the addition of the NPs. The radical scavenging activity (%) was calculated as follows: scavenging activity (%) = (1 - ABS_DPPH + NPs_ / ABS_DPPH_) × 100, where ABS_DPPH + NPs_ represents the absorbance at 517 nm of DPPH in the presence of IR780-Mn@TA-TPL NPs, while ABS_DPPH_ represents the absorbance at 517 nm of DPPH alone. H_2_O_2_ scavenging activity was also determined. Briefly, 2 μL of H_2_O_2_ (30%, Aladdin) was added to 1.98 mL of the NPs solution in phosphate buffer (pH 7.4, 0.1 M). The decrease in absorbance at 230 nm was measured using the same UV-vis spectrophotometer. The H_2_O_2_ scavenging ability was calculated using the equation: H_2_O_2_ scavenging (%) = (1 - ABS_H2O2 + NPs_ / ABS_H2O2_) × 100. Furthermore, the O_2_^•-^ and OH• scavenging ability (%) was examined using hydroxyl free radical scavenging capacity assay kit and superoxide anion content assay kit by colorimetric method (Sango Biotech, Shanghai, China) according to the manufacturer's instructions. Different concentrations of IR780-Mn@TA-TPL (10, 25, and 50 μg/mL) were added to the working solution. The absorbance at 530 nm was measured using a multiple plate reader after standing for 20 min.

### Cellular uptake

PC12 and BV2 cells were seeded at a density of 1 × 10^5^ cells in confocal dishes and cultured for 24 h. Subsequently, the cells were treated with 50 µg/mL of either IR780-Mn@TA NPs or IR780-Mn@TA-TPL NPs and co-incubated for 4 h. Following co-incubation, the cells were washed thrice with PBS, and then stained with DAPI (Thermo Fisher Scientific, USA) for 10 min. Cellular fluorescence was then examined using confocal microscopy (Leica TCS SP8, USA). For the Bio-TEM assay, PC12 cells from each treatment group were fixed with 4% paraformaldehyde (Sigma-Aldrich, USA). The fixed cells were then pipetted onto 600-mesh copper grids at a concentration range of 0.1-10 μM and allowed to adhere for 1 min. Subsequently, the grids were negatively stained with 2% uranyl acetate (Sigma-Aldrich, USA) for 45 s. The samples were dried and examined under a transmission electron microscope operating at 200 kV. To establish the* in vitro* BBB models for analyzing the internalization of NPs in PC12 cells across the *in vitro* BBB, bEnd.3 cells were seeded in the upper chambers (pore size: 0.4 μm) at 2 × 10^5^ cells/insert. The medium was changed every 2 days. After 6 days, PC12 cells were seeded on the lower chamber. After co-incubation for 24 h, the integrity of the barrier was confirmed by detecting the TEER. Monolayers with TEER over 200 Ω cm^-2^ were used for further experiments. IR780-Mn@TA-TPL and IR780-Mn@TA NPs (50 μg/mL) were added into upper inserts. The fluorescence images of NPs in PC12 ells were captured after incubation for 12 h, and 24h, respectively.

### Flow cytometry analysis of cell apoptosis

PC12 cells were plated at a density of 1 × 10^5^ cells per well in 6-well plates and incubated for 24 h. Subsequently, the cells were exposed to OA at a concentration of 50 nM for 12 h. Following the OA treatment, the experimental groups were administered either IR780-Mn@TA NPs or IR780-Mn@TA-TPL NPs at a concentration of 50 µg/mL for an additional 12 h. Meanwhile, the control and OA-treated groups had their media replaced with fresh culture medium. After treatment, cells were harvested, resuspended, and then incubated with a Propidium Iodide (PI)/Annexin V-FITC apoptosis detection kit (Beyotime Biotechnology, China) according to the manufacturer's instructions. The fluorescence intensity indicative of apoptotic events was quantified using a BD LSRFortessa flow cytometer (BD Biosciences, USA).

### Intracellular ROS scavenging activity analysis

After treatment, the cell medium was remoced and cells were washed with PBS and then incubated with 1ml of 5 µM MitoSOX reagent working solution (Invitrogen, USA) and incubated at 37 °C for 30 min. The cells were then washed with PBS and incubated with DAPI for 10 min. Confocal microscopy (Leica TCS SPE, USA) was used to evaluate the relative scavenging activity of mitochondrial ROS. In addition, intracellular total ROS accumulation was evaluated using flow cytometry after stained with DCFH-DA (5 µM) at 37 °C for 15 min. The cells were collected by centrifugation and resuspended in 500 µL PBS for analysis on BD LSRFortessa flow cytometer.

### Monitoring of tau aggregation

Recombinant tau protein solution (Abcam, USA) was mixed with IR780-Mn@TA-TPL NPs in TBS buffer solution (10 mM Tris-HCl, 30 mM NaCl, pH = 7.4). Heparin (Mw = 15,000) was added to a final concentration of 5 μM 41. The protein samples were then put on a shaker at 37 °C at 600 rpm. To monitor tau fibrillation progress, aliquots of the samples were withdrawn for measurements at indicated time points. ThS molecules displayed enhanced fluorescence intensity upon specifically bind to β-sheet structures present in fibrillar assemblies. Therefore, our study employed ThS dye was to monitor the self-assembly of tau protein, as the primary structure in tau aggregates is composed of β-sheets. For ThS assay in solution, the protein samples (10 μM, 20 μL) were added to ThS buffer solution (20 μM, 980 μL) and measured by fluorescence spectroscopy. Excitation/Emission = 488nm/500nm. For intracellular ThS staining, fixed and permeabilized PC12 cells were incubated with 0.05% ThS for 10 min and were washed 3 times with 70% ethanol, then cells were directly counterstained with DAPI for 10 min. The intracellular tau aggregates were assessed by measuring the fluorescence intensity of ThS fluorescent signals using confocal microscopy (Leica TCS SPE, USA).

### Animal modeling

Female SD rats (4-6 months old) were purchased from Centralized Animal Facilities of The Hong Kong Polytechnic University. All animal protocols were conducted under the Guidelines for Care and Use of Laboratory Animals of Department of Health of the Government of the Hong Kong Special Administrative Region. An animal ethics approval was obtained from the Animal Ethics Committee of The Hong Kong Polytechnic University (Ref No.: 20-21/325-BME-R-GRF) with an animal license issued by the Department of Health of the Government of the Hong Kong Special Administrative Region (Ref No.: (21-187) in DH/HT&A/8/2/4 Pt.6). Before surgery, rats were anesthetized with a mixture of 50 mg/kg ketamine and 10 mg/kg xylazine. Following established protocols in previous studies, OA (300 ng in 1.5 µL of saline) or saline (0.9% NaCl for medical use, 1.5 µL) was stereotaxically microinjected into the right dorsal hippocampus (AP -3.8 mm, ML -2.5 mm, and DV -3.2 mm, according to the Rat Brain Paxinos Atlas) to establish the AD model and sham groups, respectively 11, 51. The microinjection was performed using a 10 µL gas phase microsampler (RWD Life Science Co., LTD, China) for around 15 min. The needle was then held in place for an additional 10 min to allow for diffusion before being removed slowly over 5 min. Post-surgery, the rats received an intraperitoneal administration of 80 units of ibuprofen daily for 3 days to manage inflammation and pain.

### *In vivo* fluorescence imaging

IR780 fluorescent dye was introduced to the tag nanocomposite for *in vivo* fluorescence imaging. At designated time points post-treatment, rats from all experimental groups underwent imaging using an IVIS system (PerkinElmer IVIS Lumina Series III). The system was set to an excitation wavelength of 780 nm and an emission wavelength of 845 nm. Subsequent image analysis was conducted utilizing Living Image software.

### *In vivo* MR imaging

The relaxation times of IR780-Mn@TA-TPL NPs at varying concentrations, along with *in vivo* MR imaging, were acquired using a Pharmascan 70/16 US system. Spin-lattice relaxation time (T_1_) was measured using the T_1_ mapping IR-FSE sequence: TR = 1000 ms, TE =13.8 ms. Spin-spin relaxation time (T_2_) was measured using the T_2_ mapping IR-FSE sequence: TR = 2000 ms, TE = 80 ms. For* in vivo* MR imaging, IR780-Mn@TA-TPL NPs or IR780-Mn@TA NPs were administered via a unilateral hippocampal injection and then T1-weighted MR imaging was performed using an FSE sequence with the following parameters: TR = 550 ms, TE = 12 ms, field of view (FOV) = 14 mm × 14 mm, slice thickness (SL) = 2 mm, and a flip angle of 90°.

### Y maze behavioral test

Behavioral tests were performed on SD rats using a Y-maze consisting of three arms (50 cm × 16 cm × 32 cm). Seven days following the treatment, the rats underwent two days of training. During this period, they were allowed 10 min of free exploration in the Y-maze with one arm blocked. Subsequently, 24 h after the last training session, the previously closed arm was opened for the test phase. The behavior of each rat was recorded and analyzed using Smart version 3.0 software.

### Statistical analysis

Experimental data are presented as mean ± standard deviation (SD). For comparisons involving multiple groups, one-way analysis of variance (ANOVA). Statistical analyses were conducted using GraphPad Prism V9.5. A probability value (p) of less than 0.05 was considered to denote statistical significance.

## Supplementary Material

Supplementary figures.

## Abbreviations

AD: Alzheimer's disease
Aβ: amyloid-β
p-tau: hyperphosphorylated tau
TA: tannic acid
OA: okadaic acid
ROS: reactive oxygen species
MRI: magnetic resonance imaging
NFTs: neurofibrillary tangles
NPs: nanoparticles
DSPE-PEG: distearoylphosphatidylethanolamine
CMC: critical micelle concentration
TEM: transmission electron microscopy
EE: encapsulation efficiency
FTIR: fourier-transform infrared spectrum
EDS: energy-dispersive spectroscopy
PBS: phosphate-buffered saline
DLS: dynamic light scattering
DPPH: α,α diphenyl-β-picrylhydrazyl
•OH: hydroxyl radical
O_2_^•-^: superoxide anions
H_2_O_2_: hydrogen peroxide
ThS: thioflavin S
DCFH-DA: Dichlorodihydrofluorescein diacetate
ΔΨm: mitochondrial membrane potential
Bax: BCL2-associated X
IVIS: *in vivo* imaging system
ICP-MS: inductively coupled plasma mass spectrometry
H&E: hematoxylin and eosin
PFA: paraformaldehyde

## References

[1] Dubois B, Hampel H, Feldman HH, Scheltens P, Aisen P, Andrieu S (2016). Preclinical Alzheimer's disease: Definition, natural history, and diagnostic criteria. Alzheimers Dement.

[2] Wang J, Fan Y, Tan Y, Zhao X, Zhang Y, Cheng C (2018). Porphyrinic Metal-Organic Framework PCN-224 Nanoparticles for Near-Infrared-Induced Attenuation of Aggregation and Neurotoxicity of Alzheimer's Amyloid-beta Peptide. ACS Appl Mater Interfaces.

[3] Savelieff MG, Nam G, Kang J, Lee HJ, Lee M, Lim MH (2019). Development of Multifunctional Molecules as Potential Therapeutic Candidates for Alzheimer's Disease, Parkinson's Disease, and Amyotrophic Lateral Sclerosis in the Last Decade. Chem Rev.

[4] Palop JJ, Mucke L (2010). Amyloid-beta-induced neuronal dysfunction in Alzheimer's disease: from synapses toward neural networks. Nat Neurosci.

[5] Panza F, Lozupone M, Logroscino G, Imbimbo BP (2019). A critical appraisal of amyloid-beta-targeting therapies for Alzheimer disease. Nat Rev Neurol.

[6] Wang J, Gu Y, Liu X, Fan Y, Zhang Y, Yi C (2022). Near-Infrared Photothermally Enhanced Photo-Oxygenation for Inhibition of Amyloid-beta Aggregation Based on RVG-Conjugated Porphyrinic Metal-Organic Framework and Indocyanine Green Nanoplatform. Int J Mol Sci.

[7] Ferguson FM, Gray NS (2018). Kinase inhibitors: the road ahead. Nat Rev Drug Discov.

[8] Bhatt S, Puli L, Patil CR (2021). Role of reactive oxygen species in the progression of Alzheimer's disease. Drug Discov Today.

[9] Liu Z, Zhou T, Ziegler AC, Dimitrion P, Zuo L (2017). Oxidative Stress in Neurodegenerative Diseases: From Molecular Mechanisms to Clinical Applications. Oxid Med Cell Longev.

[10] Zhang Q, Li C, Yin B, Yan J, Gu Y, Huang Y (2024). A biomimetic upconversion nanoreactors for near-infrared driven H2 release to inhibit tauopathy in Alzheimer's disease therapy. Bioactive Materials.

[11] Chen Q, Du Y, Zhang K, Liang Z, Li J, Yu H (2018). Tau-Targeted Multifunctional Nanocomposite for Combinational Therapy of Alzheimer's Disease. ACS Nano.

[12] Cui X, Lin Q, Liang Y (2020). Plant-Derived Antioxidants Protect the Nervous System From Aging by Inhibiting Oxidative Stress. Front Aging Neurosci.

[13] Dare RG, Nakamura CV, Ximenes VF, Lautenschlager SOS (2020). Tannic acid, a promising anti-photoaging agent: Evidences of its antioxidant and anti-wrinkle potentials, and its ability to prevent photodamage and MMP-1 expression in L929 fibroblasts exposed to UVB. Free Radic Biol Med.

[14] Byun H, Jang GN, Hong MH, Yeo J, Shin H, Kim WJ (2022). Biomimetic anti-inflammatory and osteogenic nanoparticles self-assembled with mineral ions and tannic acid for tissue engineering. Nano Converg.

[15] He M, Yang B, Huo F, Xie L, Yang M, Tian W (2021). A novel coating with universal adhesion and inflammation-responsive drug release functions to manipulate the osteoimmunomodulation of implants. J Mater Chem B.

[16] He M, Gao X, Fan Y, Xie L, Yang M, Tian W (2021). Tannic acid/Mg(2+)-based versatile coating to manipulate the osteoimmunomodulation of implants. J Mater Chem B.

[17] Son MH, Park SW, Jung YK (2021). Antioxidant and anti-aging carbon quantum dots using tannic acid. Nanotechnology.

[18] Pucci C, Martinelli C, De Pasquale D, Battaglini M, di Leo N, Degl'Innocenti A (2022). Tannic Acid-Iron Complex-Based Nanoparticles as a Novel Tool against Oxidative Stress. ACS Appl Mater Interfaces.

[19] Yao J, Gao X, Sun W, Yao T, Shi S, Ji L (2013). Molecular hairpin: a possible model for inhibition of tau aggregation by tannic acid. Biochemistry.

[20] Hu Y, Hu X, Lu Y, Shi S, Yang D, Yao T (2020). New Strategy for Reducing Tau Aggregation Cytologically by A Hairpinlike Molecular Inhibitor, Tannic Acid Encapsulated in Liposome. ACS Chem Neurosci.

[21] Lu S, Xia R, Wang J, Pei Q, Xie Z, Jing X (2021). Engineering Paclitaxel Prodrug Nanoparticles via Redox-Activatable Linkage and Effective Carriers for Enhanced Chemotherapy. ACS Appl Mater Interfaces.

[22] Yang J, Li Q (2020). Manganese-Enhanced Magnetic Resonance Imaging: Application in Central Nervous System Diseases. Front Neurol.

[23] Fernsebner K, Zorn J, Kanawati B, Walker A, Michalke B (2014). Manganese leads to an increase in markers of oxidative stress as well as to a shift in the ratio of Fe(II)/(III) in rat brain tissue. Metallomics.

[24] Azria D, Blanquer S, Verdier JM, Belamie E (2017). Nanoparticles as contrast agents for brain nuclear magnetic resonance imaging in Alzheimer's disease diagnosis. J Mater Chem B.

[25] Qi Y, Li J, Nie Q, Gao M, Yang Q, Li Z (2021). Polyphenol-assisted facile assembly of bioactive nanoparticles for targeted therapy of heart diseases. Biomaterials.

[26] Che J, Okeke CI, Hu ZB, Xu J (2015). DSPE-PEG: a distinctive component in drug delivery system. Curr Pharm Des.

[27] Lu Y, Yue Z, Xie J, Wang W, Zhu H, Zhang E (2018). Micelles with ultralow critical micelle concentration as carriers for drug delivery. Nat Biomed Eng.

[28] Sahiner N, Sengel SB (2016). Tannic acid decorated poly(methacrylic acid) micro and nanoparticles with controllable tannic acid release and antioxidant properties. Colloids and Surfaces A: Physicochemical and Engineering Aspects.

[29] Bubpamala T, Promoppatum P, Pholpabu P (2024). Drug-Releasing Tannic Acid-Mediated Adhesive PEG Hydrogel for Porous Titanium Implants. ACS Omega.

[30] Shi P, Hu X, Wang Y, Duan M, Fang S, Chen W (2018). A PEG-tannic acid decorated microfiltration membrane for the fast removal of Rhodamine B from water. Separation and Purification Technology.

[31] Chen C, Geng XW, Pan YH, Ma YN, Ma YX, Gao SZ (2020). Synthesis and characterization of tannic acid-PEG hydrogel via Mitsunobu polymerization. RSC Adv.

[32] Li S, Johnson J, Peck A, Xie Q (2017). Near infrared fluorescent imaging of brain tumor with IR780 dye incorporated phospholipid nanoparticles. J Transl Med.

[33] Guo Q, Xu S, Yang P, Wang P, Lu S, Sheng D (2020). A dual-ligand fusion peptide improves the brain-neuron targeting of nanocarriers in Alzheimer's disease mice. J Control Release.

[34] Xu S, Yang P, Qian K, Li Y, Guo Q, Wang P (2022). Modulating autophagic flux via ROS-responsive targeted micelles to restore neuronal proteostasis in Alzheimer's disease. Bioact Mater.

[35] Huang Y, Zhang Q, Lam CYK, Li C, Yang C, Zhong Z (2024). An Aggregation-Induced Emission-Based Dual Emitting Nanoprobe for Detecting Intracellular pH and Unravelling Metabolic Variations in Differentiating Lymphocytes. ACS Nano.

[36] Zhang Q, Yin B, Hao J, Ma L, Huang Y, Shao X An AIEgen/graphene oxide nanocomposite (AIEgen@GO)-based two-stage "turn-on" nucleic acid biosensor for rapid detection of SARS-CoV-2 viral sequence. Aggregate. 2022: e195.

[37] Gao H, Xiu MQ, Wang MY, Zhan BY, Deng X, Xu Y (2019). Systematic Investigation on the Adsorption Performance and Mechanism of MnO2/TA Nanoflowers for Cu(II) Removal from Aqueous Solution. ChemistrySelect.

[38] Sadat SMA, Jahan ST, Haddadi A (2016). Effects of Size and Surface Charge of Polymeric Nanoparticles on *in Vitro* and *in Vivo* Applications. Journal of Biomaterials and Nanobiotechnology.

[39] Gulcin İ, Alwasel SH (2023). DPPH Radical Scavenging Assay. Processes.

[40] Giovannini J, Smeralda W, Jouanne M, Sopkova-de Oliveira Santos J, Catto M, Voisin-Chiret AS (2022). Tau protein aggregation: Key features to improve drug discovery screening. Drug Discov Today.

[41] Zhu L, Xu L, Wu X, Deng F, Ma R, Liu Y (2021). Tau-Targeted Multifunctional Nanoinhibitor for Alzheimer's Disease. ACS Appl Mater Interfaces.

[42] Ji W, Li Y, Peng H, Zhao R, Shen J, Wu Y (2022). Self-Catalytic Small Interfering RNA Nanocarriers for Synergistic Treatment of Neurodegenerative Diseases. Adv Mater.

[43] Zhang Q, Yin B, Huang Y, Gu Y, Yan J, Chen J (2023). A dual "turn-on" biosensor based on AIE effect and FRET for *in situ* detection of miR-125b biomarker in early Alzheimer's disease. Biosens Bioelectron.

[44] Samluk L, Ostapczuk P, Dziembowska M (2022). Long-term mitochondrial stress induces early steps of Tau aggregation by increasing reactive oxygen species levels and affecting cellular proteostasis. Mol Biol Cell.

[45] Misrani A, Tabassum S, Yang L (2021). Mitochondrial Dysfunction and Oxidative Stress in Alzheimer's Disease. Front Aging Neurosci.

[46] Rizwan H, Pal S, Sabnam S, Pal A (2020). High glucose augments ROS generation regulates mitochondrial dysfunction and apoptosis via stress signalling cascades in keratinocytes. Life Sci.

[47] Zhang L, Zhao P, Yue C, Jin Z, Liu Q, Du X (2019). Sustained release of bioactive hydrogen by Pd hydride nanoparticles overcomes Alzheimer's disease. Biomaterials.

[48] Taylor RC, Cullen SP, Martin SJ (2008). Apoptosis: controlled demolition at the cellular level. Nat Rev Mol Cell Biol.

[49] Sun H, Zhong Y, Zhu X, Liao H, Lee J, Chen Y (2021). A Tauopathy-Homing and Autophagy-Activating Nanoassembly for Specific Clearance of Pathogenic Tau in Alzheimer's Disease. ACS Nano.

[50] Sachdeva AK, Chopra K (2015). Naringin mitigate okadaic acid-induced cognitive impairment in an experimental paradigm of Alzheimer's disease. Journal of Functional Foods.

[51] Kamat PK, Tota S, Saxena G, Shukla R, Nath C (2010). Okadaic acid (ICV) induced memory impairment in rats: a suitable experimental model to test anti-dementia activity. Brain Res.

[52] Yang L, Wu C, Parker E, Li Y, Dong Y, Tucker L (2022). Non-invasive photobiomodulation treatment in an Alzheimer Disease-like transgenic rat model. Theranostics.

[53] Hamidi N, Nozad A, Sheikhkanloui Milan H, Amani M (2019). Okadaic acid attenuates short-term and long-term synaptic plasticity of hippocampal dentate gyrus neurons in rats. Neurobiol Learn Mem.

