# Supplementary material for: Tau‑targeting multifunctional nanocomposite based on tannic acid-metal for near-infrared fluorescence/magnetic resonance bimodal imaging-guided combinational therapy in Alzheimer's disease
Source: Theranostics. 2024 Sep 30;14(16):6218–35. doi: 10.7150/thno.98462 (PMC11488108; doi:10.7150/thno.98462)
Supplement: Supplementary file 1 — Supplementary figures. [file thnov14p6218s1.pdf]

Supporting Information

Tau-targeting multifunctional nanocomposite based on tannic acid-metal for near-infrared fluorescence/magnetic resonance bimodal imaging-guided combinational therapy in Alzheimer's disease

Yutian Gu[1,2,3,4], Qin Zhang[1,2*], Honglin Huang[1,5], Kwun Hei Willis Ho[1], Yu Zhang[6], Changqing Yi[7], Yifan Zheng[8], Raymond Chuen Chung Chang[9], Emma Shujun Wang[1], Mo Yang[1,2,3,4*]

1. Department of Biomedical Engineering, The Hong Kong Polytechnic University, Hong Kong 999077, China

2. The Hong Kong Polytechnic University Shenzhen Research Institute, Shenzhen 518000, China

3. Research Center for Nanoscience and Nanotechnology, The Hong Kong Polytechnic University, Kowloon, Hong Kong 999077, China

4. Joint Research Center of Biosensing and Precision Theranostics, The Hong Kong Polytechnic University, Kowloon, Hong Kong 999077, China

5. Department of Electrical and Electronic Engineering, The University of Hong Kong, Hong Kong 999077, China

6. Department of Mechanical and Automotive Engineering, Royal Melbourne Institute of Technology, Melbourne VIC 3000, Australia

7. Guangdong Provincial Engineering and Technology Center of Advanced and Portable Medical Devices, School of Biomedical Engineering, Sun Yat-Sen University, Shenzhen 518107, China

8. Department of Neurology, The First Affiliated Hospital, Sun Yat-sen University, Guangdong Provincial Key Laboratory of Diagnosis and Treatment of Major Neurological Diseases, National Key Clinical Department and Key Discipline of Neurology, Guangzhou 510080, China

9. School of Biomedical Sciences, LKS Faculty of Medicine, The University of Hong Kong, Hong Kong 999077, China

***Corresponding Authors:** Qin Zhang: qin7zhang@polyu.edu.hk Mo Yang: mo.yang@polyu.edu.hk

Table of Contents

Fig. S1, S2......5

Fig. S3......6

Fig. S4, S5......7

Fig. S6, S7......8

Fig. S8, S9......9

Fig. S10, S11......10

Fig. S12, S13......11

Fig. S14......12

Fig. S15, S16, S17......13

Fig. S18......14

Fig. S19......15

Fig. S20......16

Fig. S21, S22......17

Methods

Characterization TEM and EDS analyses were conducted using a FEI Tecnai F20 (FEI, USA) at an acceleration voltage of 200 kV to characterize the morphology and elemental distribution of the nanoparticles. FTIR spectroscopy measurements were obtained with a Bruker Vertex 70 FT-IR spectrometer (Rheinstetten, Germany) to confirm the successful modification of the IR780-Mn@TA-TPL NPs. Quantification of Mn content in brain tissue was carried out via ICP-MS using an Agilent 7700 series ICP-MS (USA).

Cell Culture and Cell Viability Assay In this study, PC12 cells were cultured in DMEM medium (Thermofisher, USA) containing 10% fetal bovine serum (Thermofisher, USA) and 1% penicillin-streptomycin (Invitrogen, USA) at 37 °C in 5% CO_2. Cell viability was assessed using the CCK-8 kit (Beyotime, China). To prepare IR780-Mn@TA-TPL NPs and IR780-Mn@TA NPs, the NPs were dissolved and diluted in the culture medium. Cells were seeded into 96-well plates (1×10^4/well) and incubated for 24 h before treatment with different concentrations (10-200 nM) of OA (Sigma-Aldrich, USA) for 12 h to determine the appropriate concentration range. To investigate the biocompatibility, PC12 cells were treated with sequential concentrations (0, 10, 20, 50, 100, 200, 500 µg/mL) of either IR780-Mn@TA-TPL NPs or IR780-Mn@TA NPs for 12 h. After an additional 4 h of incubation at 37 °C, the cells were subjected to the CCK-8 kit and the final optical density was recorded using a microplate reader at a wavelength of 450 nm.

Western Blotting Following treatment, cells were washed with PBS and lysed on ice using RIPA buffer (Invitrogen, USA) supplemented with 1% phosphatase inhibitor at a volume of 300 µL per dish for 30 min. The lysates were then centrifuged at 17,000 g for 15 min, and the supernatant was collected. Proteins within the supernatant were denatured by heating at 95°C for 10 min and subsequently resolved by 10% SDS-PAGE. The resolved proteins were transferred onto polyvinylidene difluoride (PVDF) membranes (Millipore, MA, USA). The membranes were blocked with 1% bovine serum albumin (BSA) for 1 h at room temperature. Overnight incubation at 4°C followed with primary antibodies targeting p-tau at Ser396, total tau, Bax, c-Caspase3, p-AKT(Ser473), total Akt, p-GSK3β

(Ser9),total GSK3β, β-actin at a dilution of 1:1000 at a dilution of 1:1000. Then, the membranes were washed and incubated with appropriate horseradish peroxidase-conjugated secondary antibodies at a dilution of 1:2500 for 2 h at room temperature before ECL imaging. The quantification of the bands was performed ImageJ software.

Immunofluorescence staining Cells from each experimental group were fixed with 4% paraformaldehyde (PFA) for 15 min, followed by permeabilization with a solution containing 0.1% Triton X-100 in PBS for 5 min. The permeabilized cells were then incubated in immunostaining blocking buffer (Beyotime, China) for 30 min at 37°C. Subsequently, the cells were cultured with primary p-tau antibody at a 1:500 dilution overnight at 4°C. After primary antibody incubation, cells were washed and incubated with the corresponding secondary antibodies, also at a 1:500 dilution, for 2 h at room temperature. Finally, cells were stained with DAPI for 10 minutes to visualize nuclei. Fluorescence signals were captured using a Leica TCS SPE confocal microscope (Leica Microsystems, USA).

Isothermal Titration Calorimetry Analysis. Isothermal titration calorimetry (ITC) was conducted at 37°C using a Malvern MicroCal PEAQ-ITC. Briefly, recombinant tau protein solution was dissolved in Tris-HCl buffer. For the ITC experiments, the tau protein in the 400 μL sample cell was adjusted to a concentration of 0.1 mM. The syringe contained the NPs solution of 2 mM. The initial drop was set to 0.4 μL, followed by titration of the IR780-Mn@TA-TPL NPs solution into the sample cell in 19 injections of 2 μL each. The interval between injections was set to 120 s to ensure complete equilibration. Raw data were recorded as a plot of heating rate (μcal/s) versus titration time (mins). Thermodynamic parameters, including stoichiometry (N), association constant (Ka), and change in enthalpy (ΔH), were obtained by nonlinear least-squares fitting of the experimental data using a single-site binding model. The binding affinity of NPs for the tau aggregates was expressed as the dissociation constant (Kd = 1/Ka).

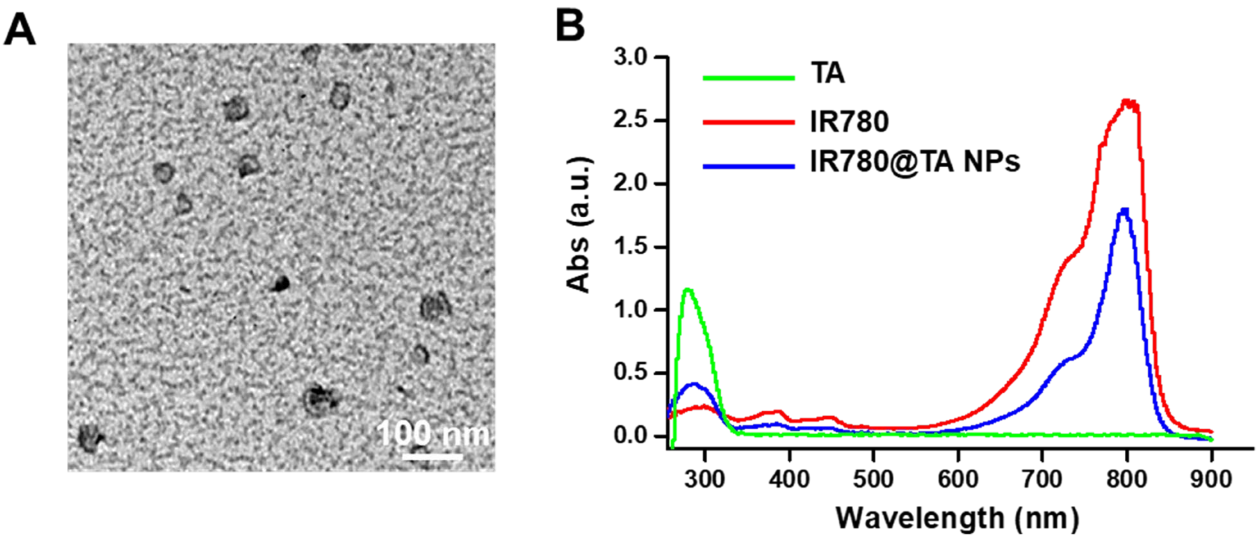

Figure S1. (A) TEM images of IR780@TA NPs. (B) The UV-Vis spectra of IR780, TA and IR780@TA NPs.

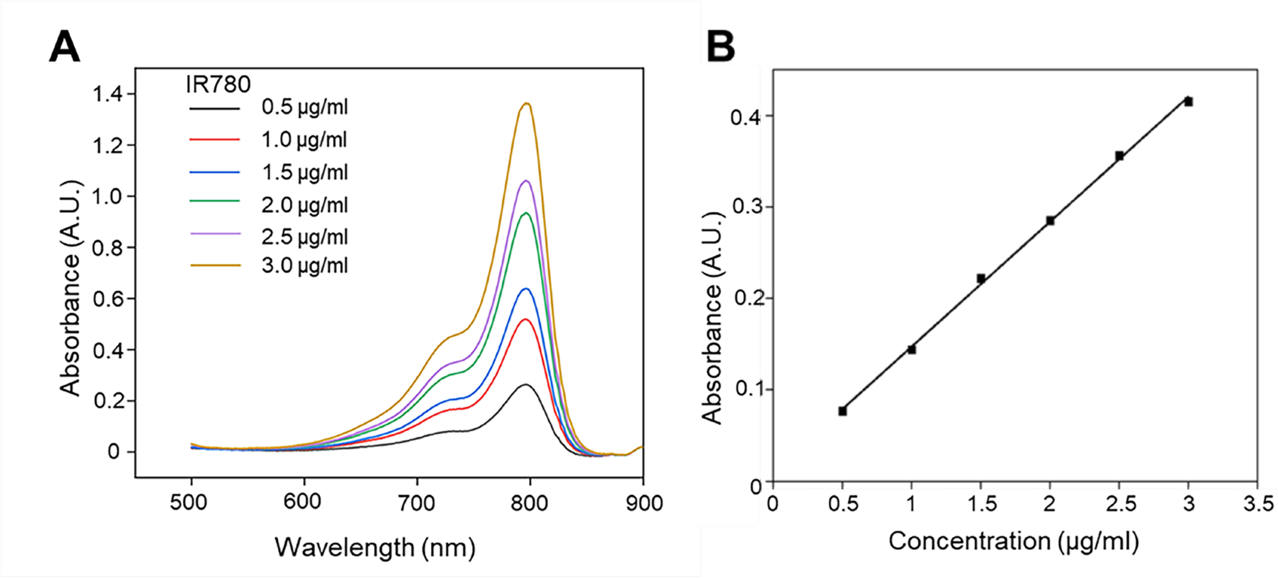

Figure S2. (A) UV-vis absorbance spectra and (B) the standard work curve of IR780 at different concentrations in DMSO.

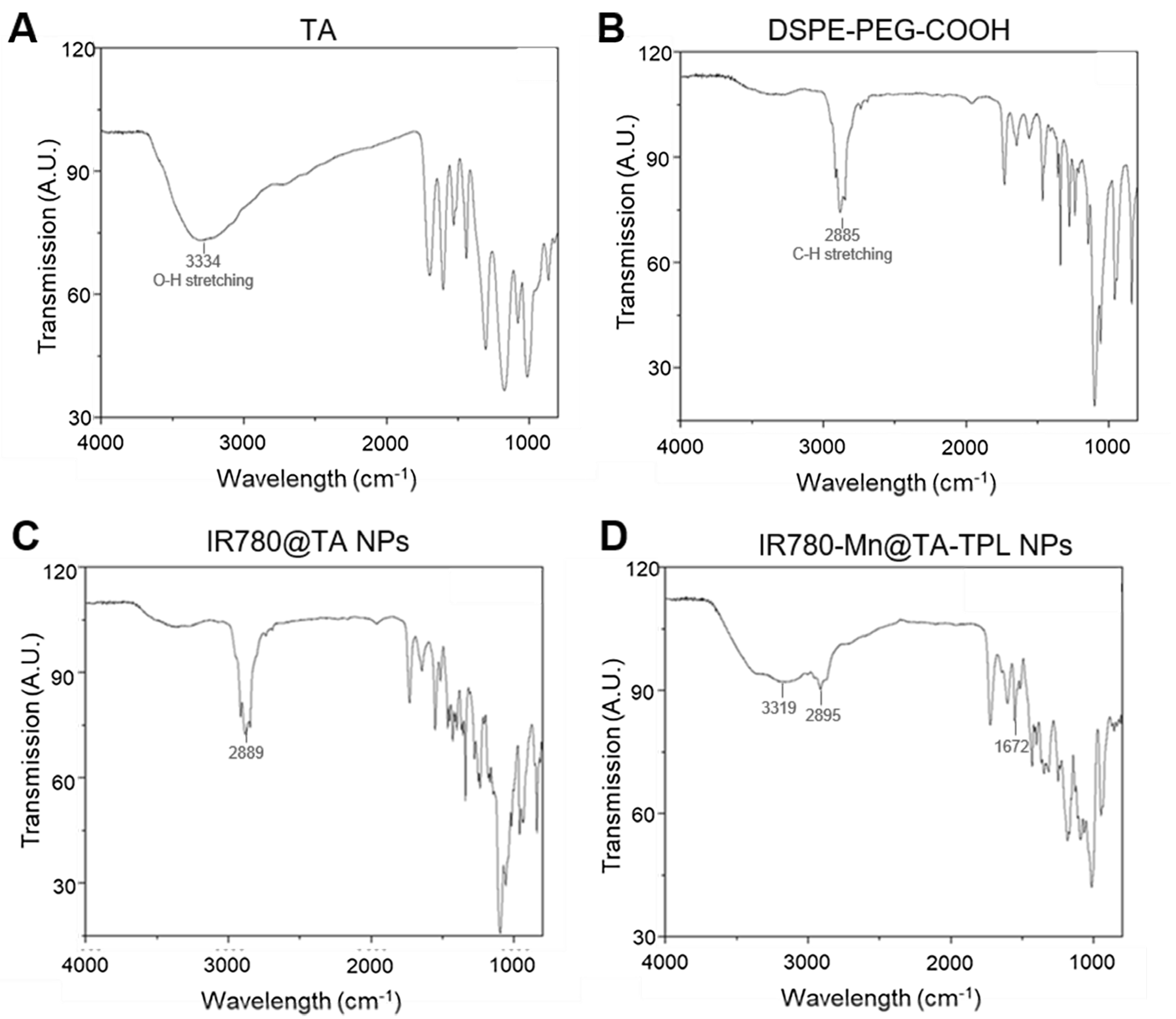

Figure S3. FTIR spectra of (A) TA, (B) DSPE-PEG-COOH, (C) IR780@TA NPs and (D) IR780-Mn@TA-TPL NPs.

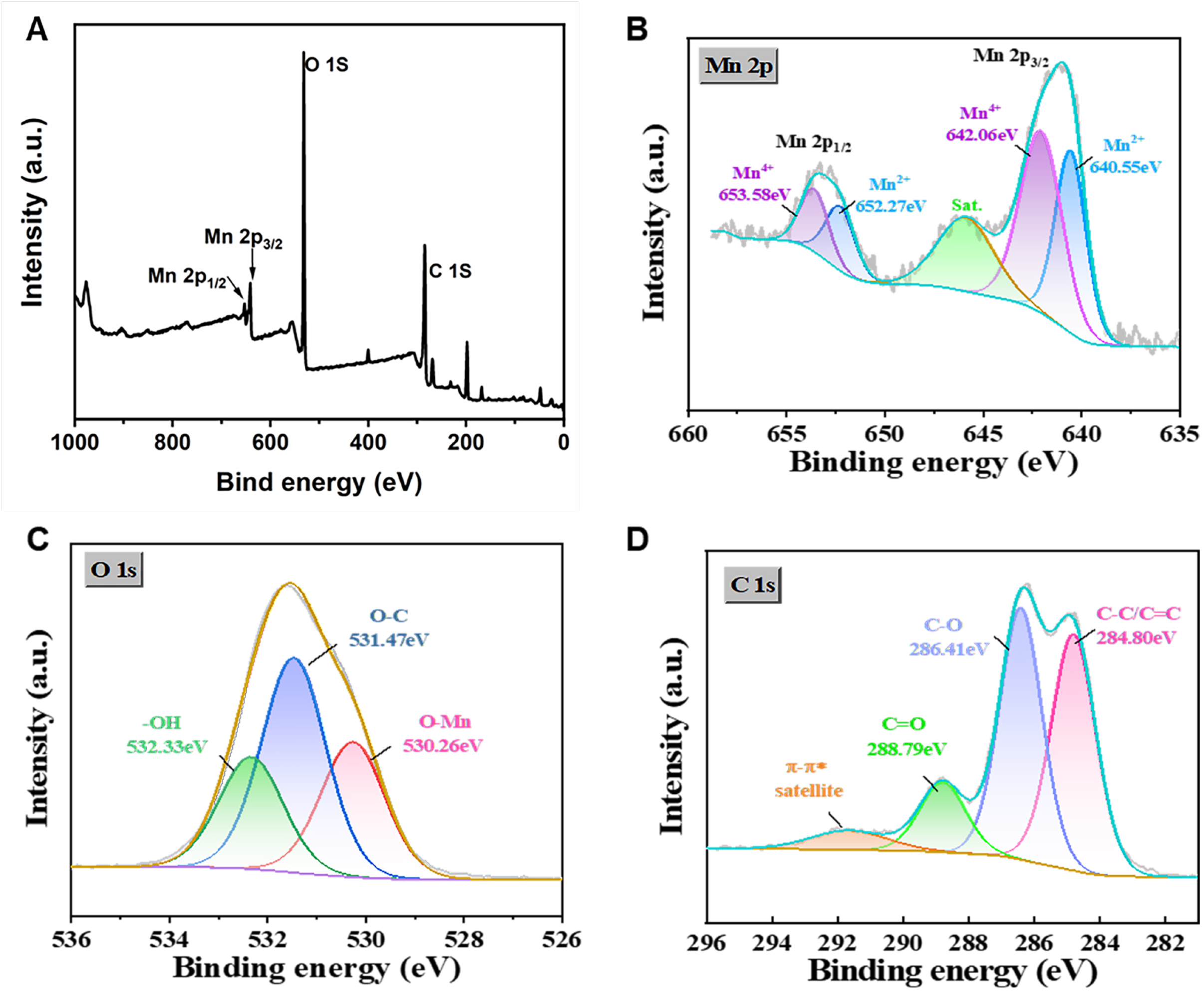

Figure S4 (A) Representative X-ray photoelectron spectroscopy (XPS) wide scan for IR780-Mn@TA NPs. High-resolution XPS spectra of (B) Mn 2p, (C) O 1s and (D) C 1s of IR780-Mn@TA NPs.

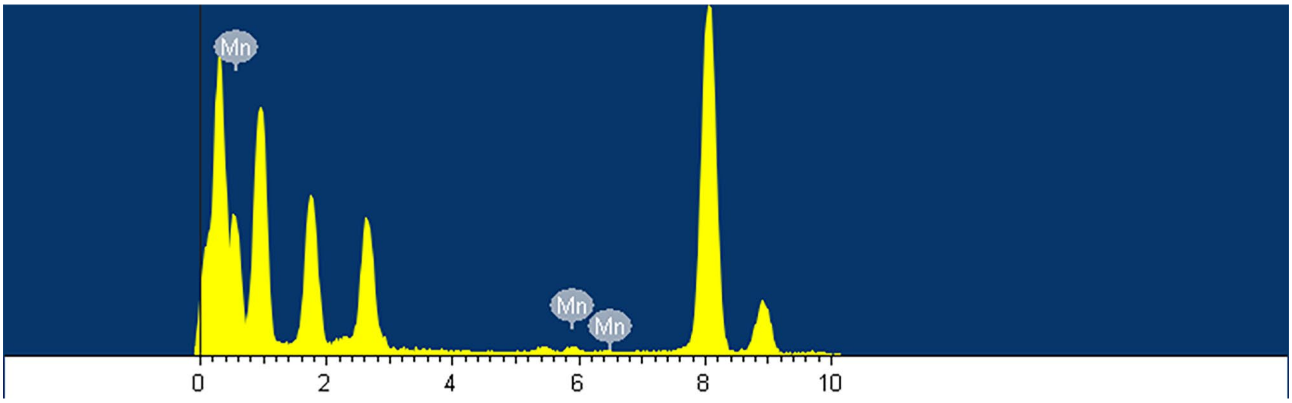

Figure S5. Energy-dispersive X-ray spectroscopy of IR780-Mn@TA NPs.

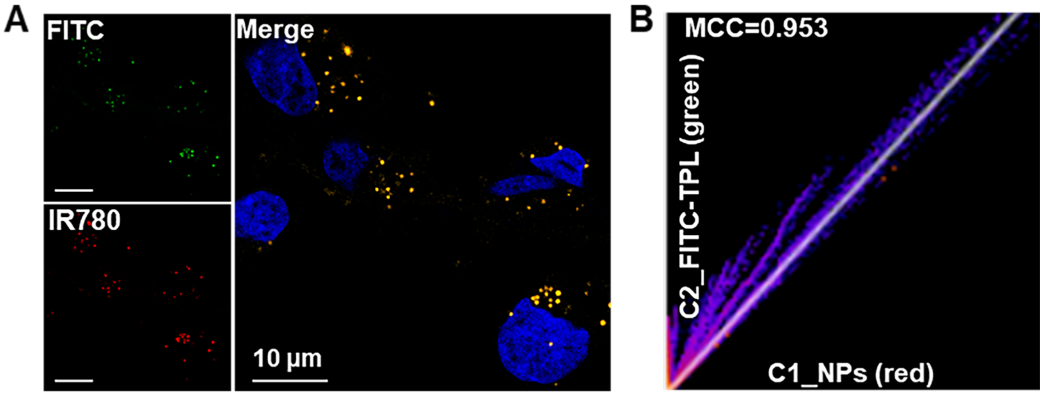

Figure S6. Characterization of the modification with FITC-TPL peptide on the surface of IR780-Mn@TA-TPL NPs. (A) The representative confocal images of IR780-Mn@TA-TPL NPs after uptake by PC12 cells. Green FL: FITC, Red FL: IR780 signals of NPs, Blue FL: DAPI-labeled cell nucleus. Scale bar:10 μm. (B) Overlap analysis of FITC-TPL and IR780 signals of the NPs by calculating the Manders' colocalization coefficient (MCC) via ImageJ software.

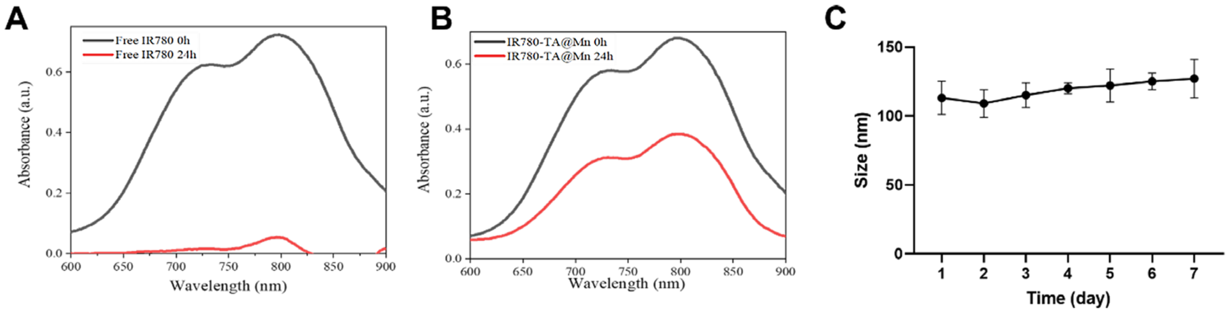

Figure S7. UV-Vis spectra of (A) free IR780 and (B) IR780-Mn@TA-TPL NPs before and after exploring in the light for 24 h. (C) The size distribution of IR780-Mn@TA-TPL NPs dispersed in PBS by DLS.

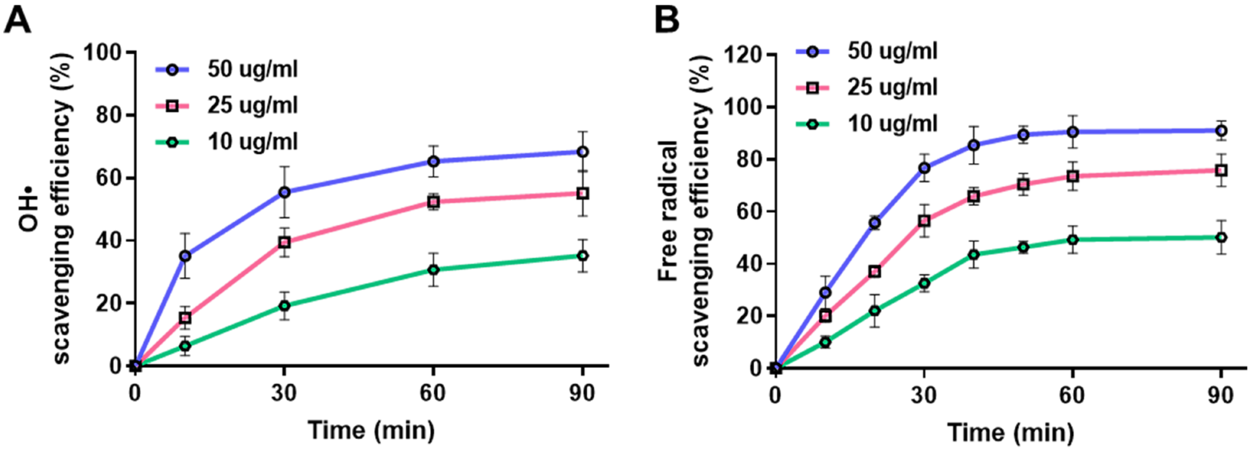

Figure S8. (A) •OH scavenging activity (%) of different concentrations of IR780-Mn@TA-TPL NPs (10, 25 and 50 μg/mL). (B) Free radical scavenging activity of IR780-Mn@TA-TPL NPs evaluated with the DPPH assay at different time points.

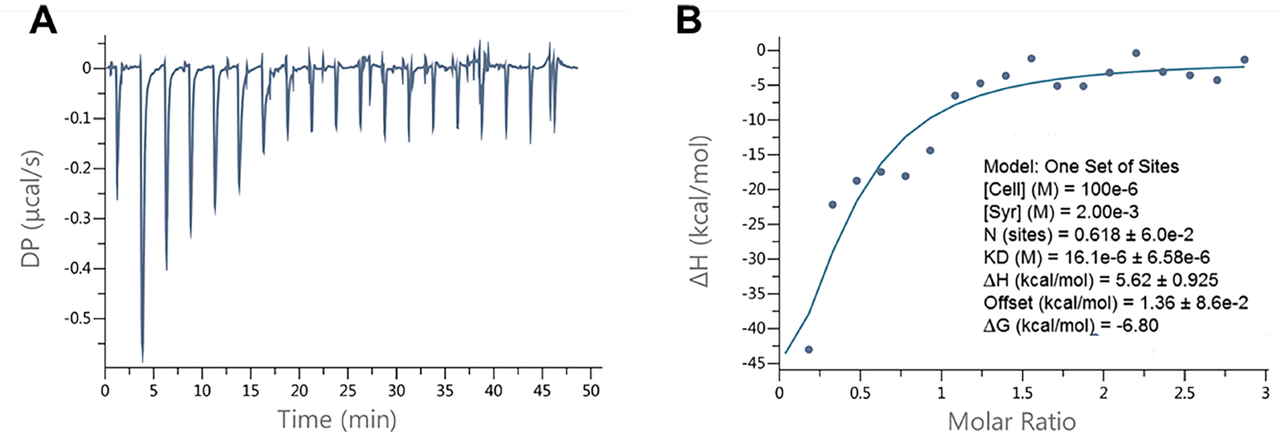

Figure S9. Experimental calorimetric data for the binding of IR780-Mn@TA-TPL NPs to tau aggregates. (A) Isothermal titration calorimetry (ITC) profiles of titration of 2 mM IR780-Mn@TA-TPL NPs to 0.1 mM tau aggregates. Time integration of the thermal power yields the heat of injection, which is plotted vs the molar ratio of NPs to tau aggregates in (B). The solid lines in the (B) represent the least-squares fitting of the data to a one-site binding model. Kd = 16.1 μM.

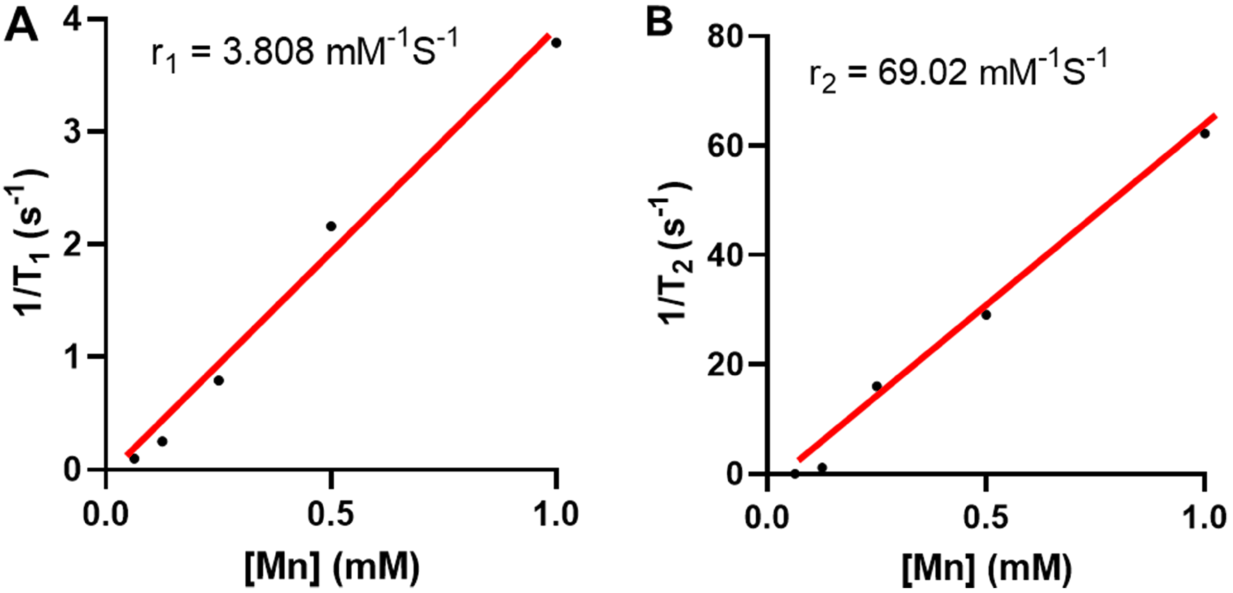

Figure S10. (A) Plot of $1/T_1$ over Mn ion concentration of $MnCl_2$.The slope indicates the specific reflexivity (r_1). (B) Plot of $1/T_2$ over Mn ion concentration of $MnCl_2$. The slope indicates the specific reflexivity (r_2).

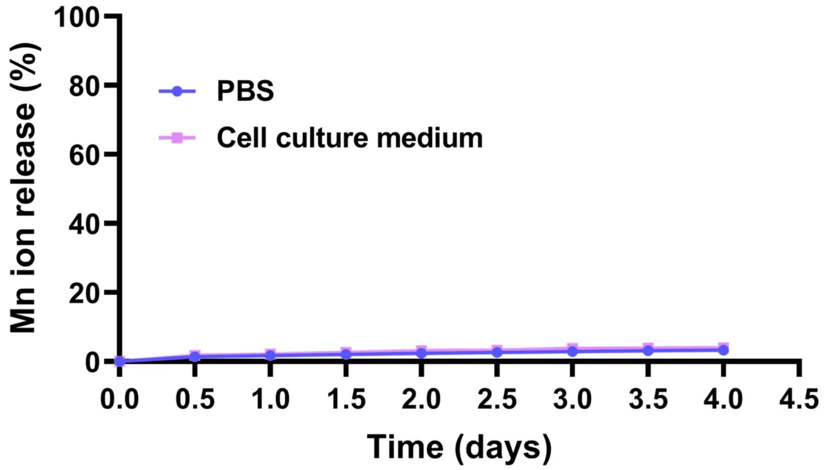

Figure S11. The Mn ions release of IR780-Mn@TA-TPL NPs storing in PBS buffer and in the cell culture medium (DEME + 10% FBS) during incubation measured by ICP-MS analysis.

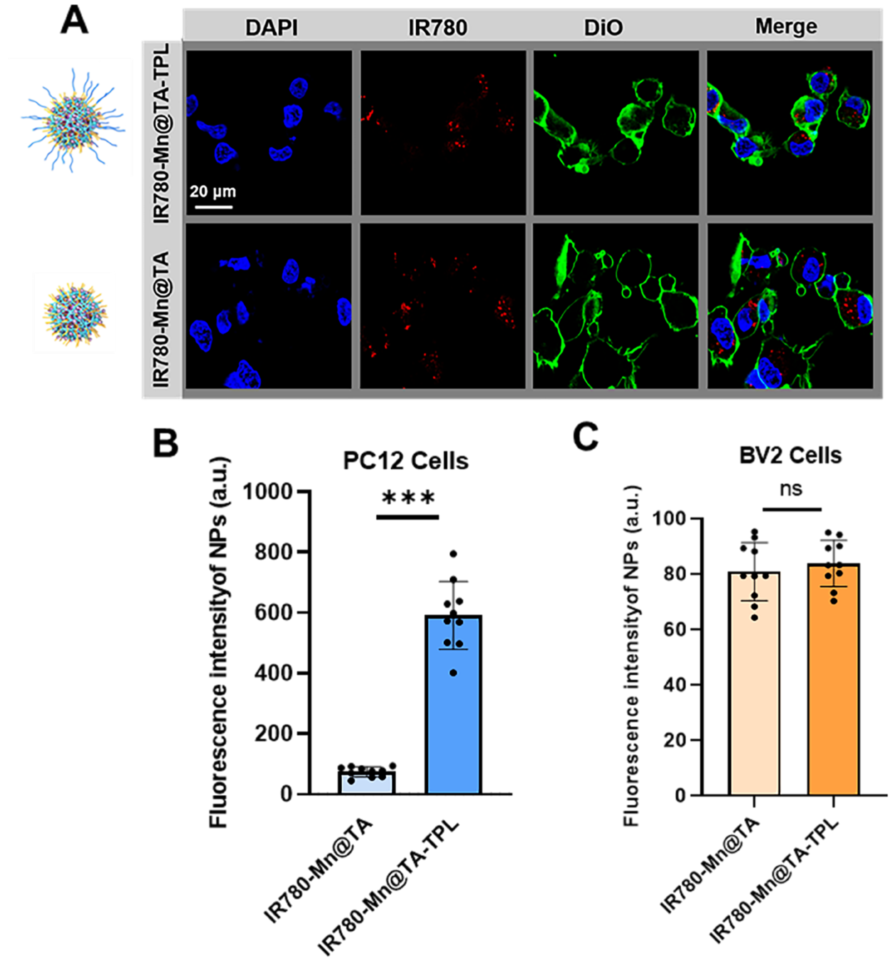

Figure S12. (A) Confocal microscopic images of BV2 cells after incubation with IR780-Mn@TA and IR780-Mn@TA-TPL NPs. DAPI: λ_{ex} = 405, λ_{em} = 420-450 nm, red fluorescence of NPs: λ_{ex} = 775 nm, λ_{em} = 790-820 nm. The quantitative analysis of the intracellular fluorescence intensity of nanoparticles (IR780-Mn@TA-TPL or IR780-Mn@TA) after uptake by (B) PC12 and (C) BV2 cells. Data were presented as mean ± SD. Significance: *** $p < 0.001$, ns: no significance. Scale bar: 20 μm.

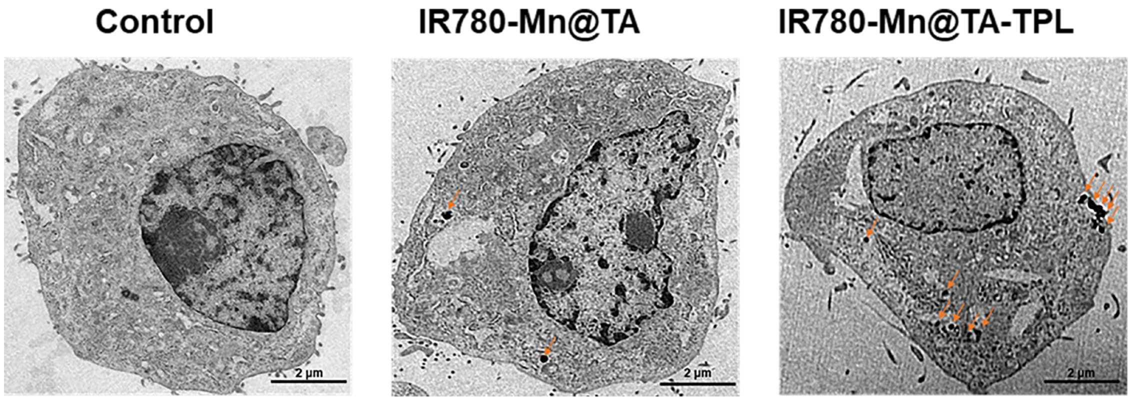

Figure S13. Bio-TEM images of PC12 cells after incubation with IR780-Mn@TA and IR780-Mn@TA-TPL NPs for 4 h.

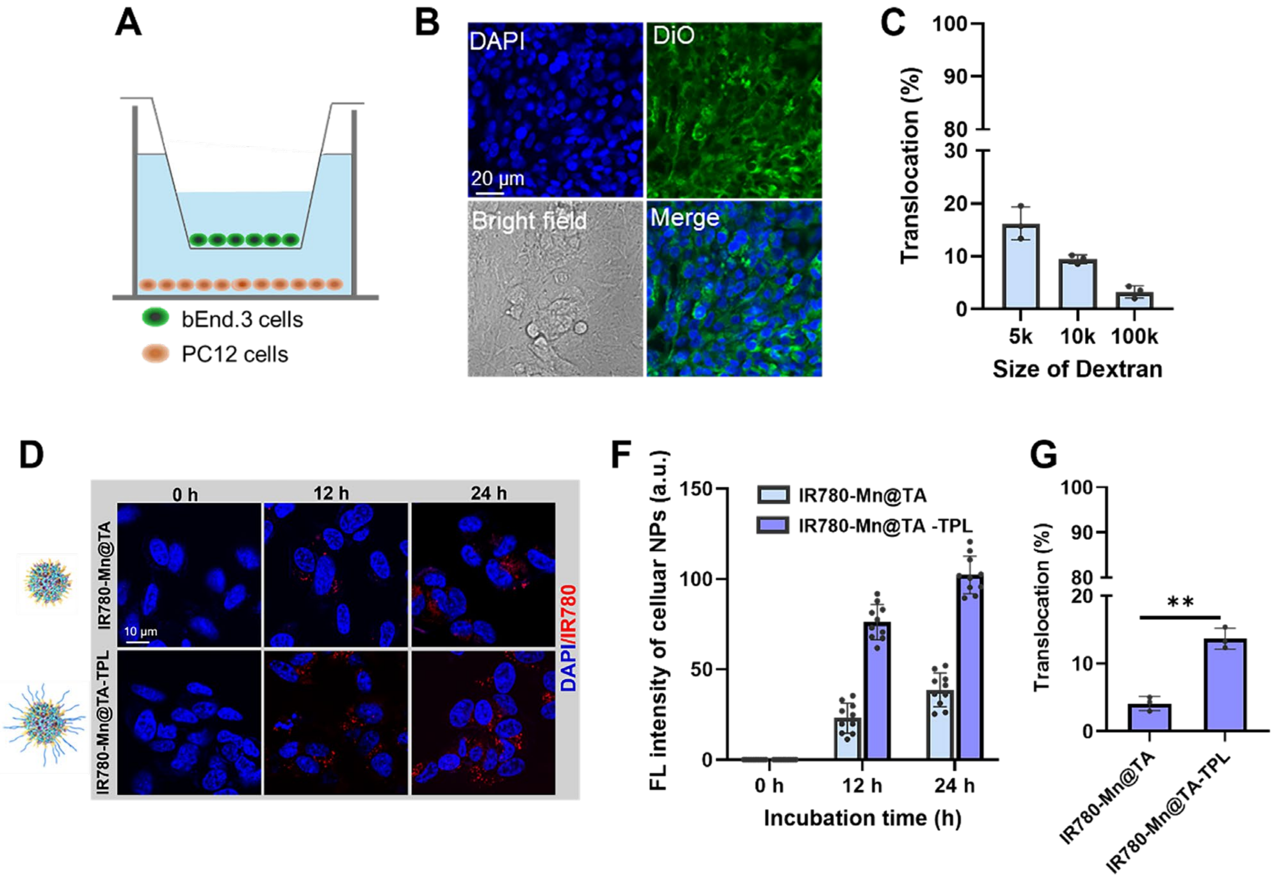

Figure S14. Evaluation of the BBB penetration ability of IR780-Mn@TA-TPL NPs by the transwell culture method. (A) Scheme of in vitro BBB transwell model. (B) Representative image of adjacent bEnd.3 cells morphology in the apical layer of BBB model. (C) The evaluation of the integrity of BBB model by measuring the fluorescence intensity of the FITC-conjugated dextran polymer (5, 10, and 100 kDa). (D) The representative fluorescence image of NPs in PC12 cells after adding IR780-Mn@TA and IR780-Mn@TA-TPL NPs into the apical side of the BBB model. (F) The mean fluorescence intensity of NPs in PC12 cells in the Transwell model, measured by Image J. The data were presented as mean ± SD. (G) IR780-Mn@TA and IR780-Mn@TA-TPL NPs was added to the apical side of the BBB model and total volume in the basolateral side collected after 24 h of incubation. The translocation rate was quantified by measuring the ratio of the fluorescence intensity in the basolateral side to the total fluorescence intensity of the NPs. Significance: ** $p < 0.01$.

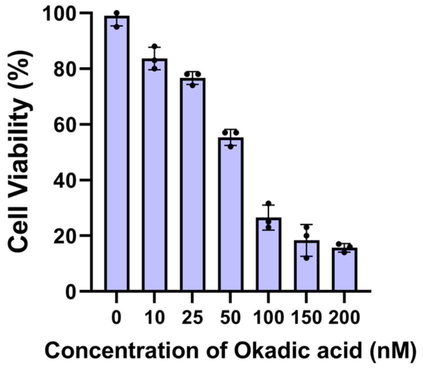

Figure S15. The relative cell viability of PC12 cells after treated with okadaic acid (OA) of different concentrations for 12 h. Data were presented as mean ± SD (n=3).

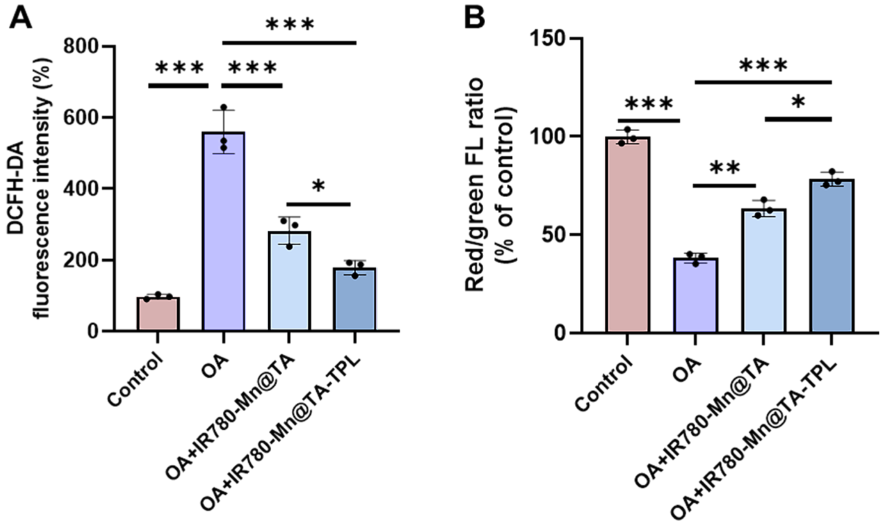

Figure S16. (A) Statistical results of intracellular fluorescence intensity of DCFH-DA measured by flow cytometry. (B) The quantitative results of red JC-1 aggregates and green monomers of OA-damaged PC12 cells after being treated with IR780-Mn@TA and IR780-Mn@TA-TPL NPs. Data were presented as mean ± SD. Significance: * $p < 0.05$, ** $p < 0.01$ and *** $p < 0.001$.

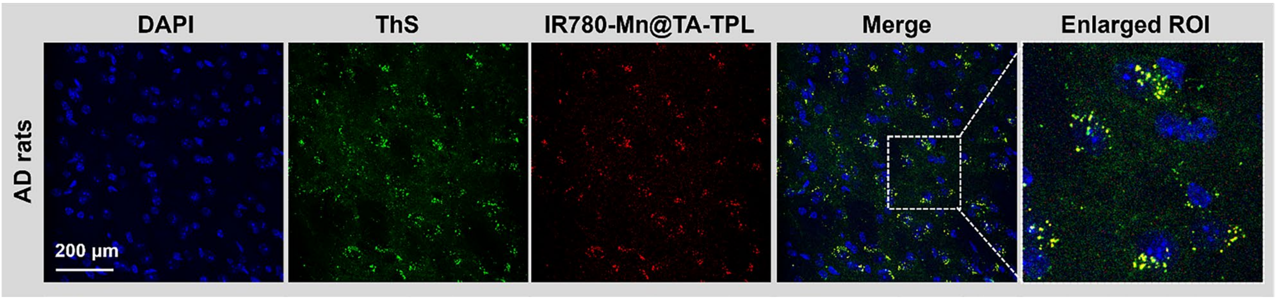

Figure S17. Representative fluorescence images of NPs (red) and ThS-stained tau aggregates (green) in the brain region of AD rats after injection with IR780-Mn@TA-TPL NPs for 4 h.

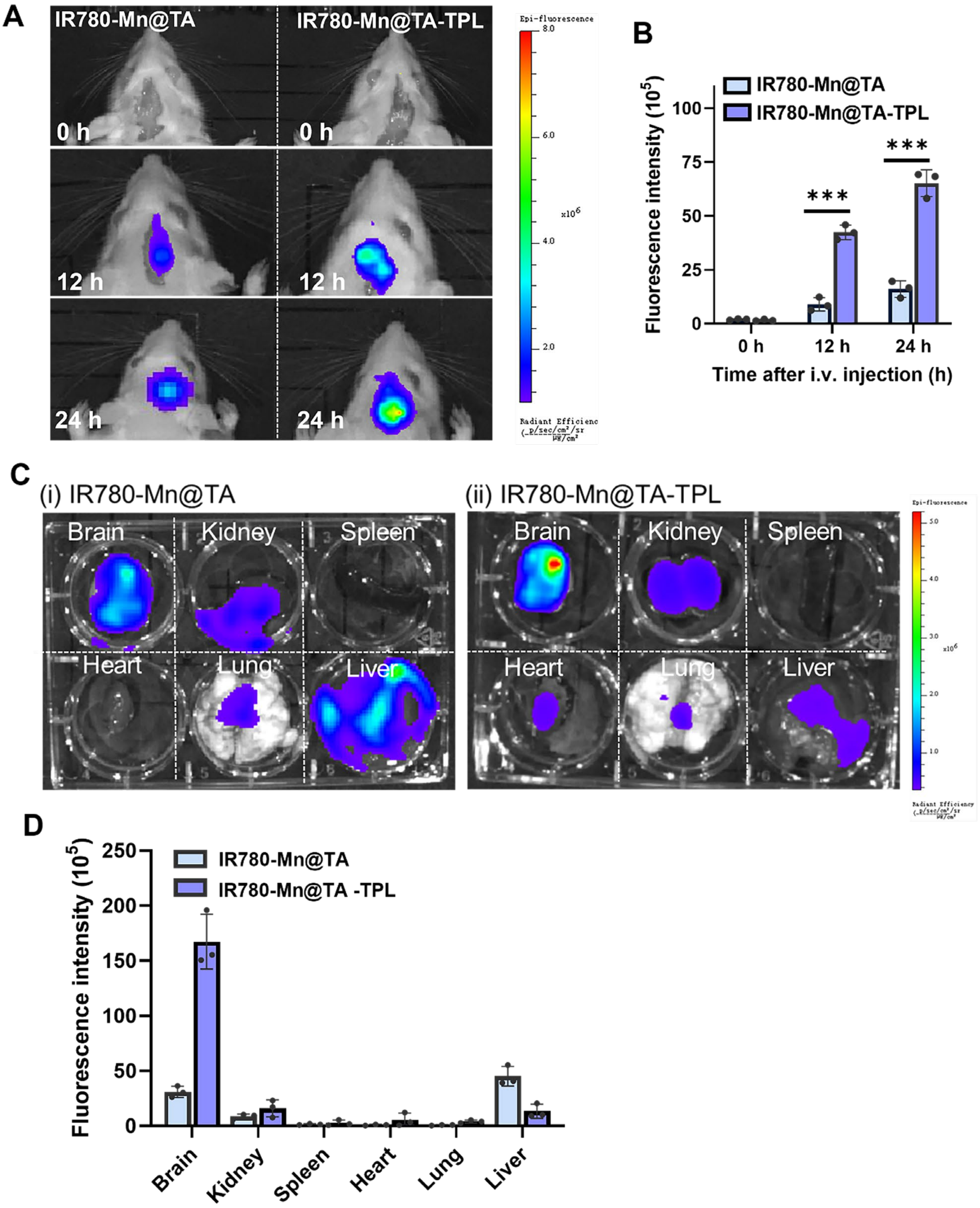

Figure S18. Evaluation of the BBB penetration ability of IR780-Mn@TA-TPL NPs in vivo. (A) IVIS imaging and (D) fluorescence intensity analysis of rat brain after intravenous injection of IR780-

Mn@TA and IR780-Mn@TA-TPL NPs at 12 h and 24 h. The data were presented as mean ± SD. (C) Ex vivo IVIS imaging of various organs after intravenous injection of IR780-Mn@TA and IR780-Mn@TA-TPL NPs for 24 h. (D) The quantitative results of the fluorescence intensity in various organ in (C). The data were presented as mean ± SD.

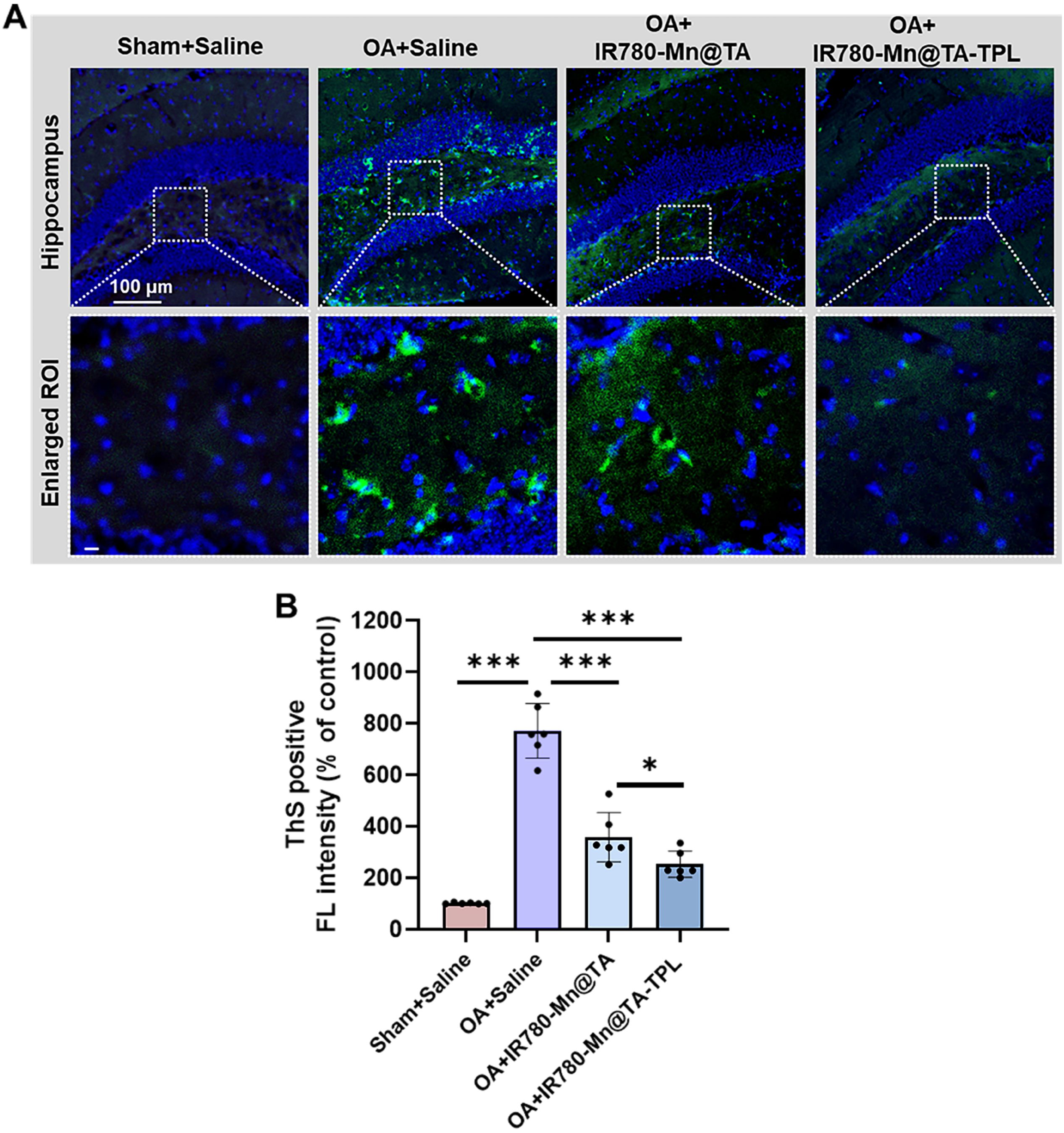

Figure S19. (A) ThS staining of the tau aggregation in the hippocampus of brain slices. (B) Quantitative results of ThS positive fluorescent intensity in the hippocampus. Data were presented as

mean ± SD. Significance: *p < 0.05 and *** p < 0.001 (n=6 per group).

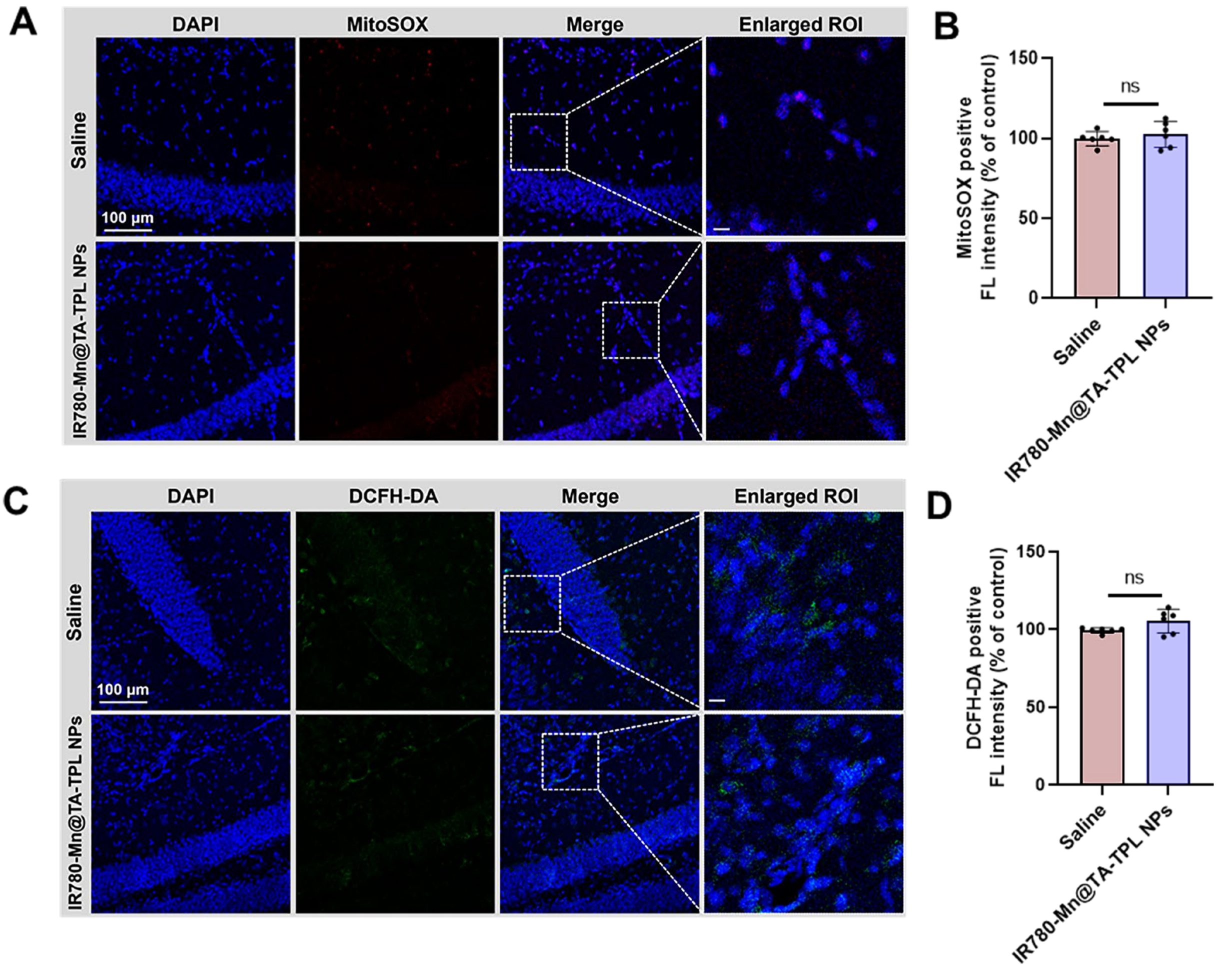

Figure S20. Representative images of (A) MitoSOX and (C) DCFH-DA staining in the hippocampus of rats after injection with saline and IR780-Mn@TA-TPL NPs. Scale bar: 100 μm. Quantitative analysis of (B) MitoSOX and (D) DCFH-DA fluorescence intensity via ImageJ software. Data were presented as mean ± SD. Significance: ns, no significance.

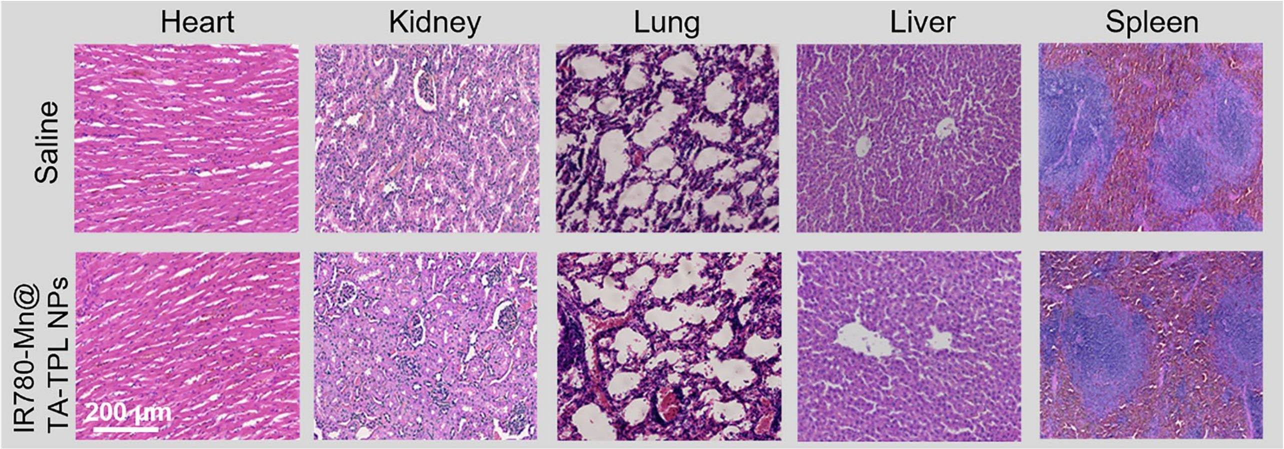

Figure S21. H&E-staining of major organs of rats after injection with saline and IR780-Mn@TA-TPL NPs. Scale bar: 200 μm.

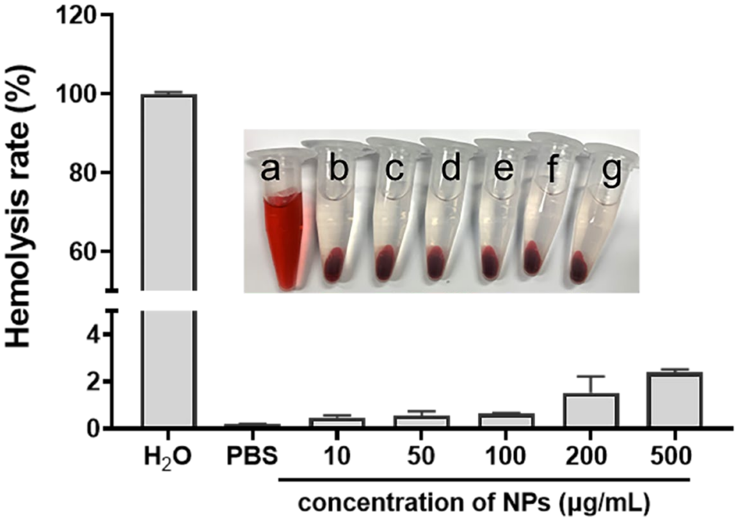

Figure S22. Hemolysis rate of IR780-Mn@TA-TPL NPs. Relative hemolysis rate in rat RBCs upon incubation with (a) H_2O as the positive control, (b) PBS as the negative control, and IR780-Mn@TA-TPL NPs at a series of concentrations, including (c) 10, (d) 50, (e) 100, (f) 200, and (g) 500 μg/mL.
